# Transfer RNA acetylation regulates in vivo mammalian stress signaling

**DOI:** 10.1126/sciadv.ads2923

**Published:** 2025-03-19

**Authors:** Supuni Thalalla Gamage, Roxane Khoogar, Shereen Howpay Manage, Judey T. DaRos, McKenna C. Crawford, Joe Georgeson, Bogdan V. Polevoda, Chelsea Sanders, Kendall A. Lee, Kellie D. Nance, Vinithra Iyer, Anatoly Kustanovich, Minervo Perez, Chu T. Thu, Sam R. Nance, Ruhul Amin, Christine N. Miller, Ronald J. Holewinski, Sudipto Das, Thomas J. Meyer, Vishal Koparde, Acong Yang, Parthav Jailwala, Joe T. Nguyen, Thorkell Andresson, Kent Hunter, Shuo Gu, Beverly A. Mock, Elijah F. Edmondson, Simone Difilippantonio, Raj Chari, Schraga Schwartz, Mitchell R. O’Connell, Colin Chih-Chien Wu, Jordan L. Meier

**Affiliations:** ^1^ Chemical Biology Laboratory, Center for Cancer Research, National Cancer Institute, National Institutes of Health, Frederick, MD, USA.; ^2^ RNA Biology Laboratory, Center for Cancer Research, National Cancer Institute, National Institutes of Health, Frederick, MD, USA.; ^3^ Department of Molecular Genetics, Weizmann Institute of Science, Rehovot 76100, Israel.; ^4^ Department of Biochemistry and Biophysics, Center for RNA Biology, School of Medicine and Dentistry, University of Rochester, Rochester, NY, USA.; ^5^ Animal Research Technical Support, Laboratory Animal Sciences Program, Frederick National Laboratory for Cancer Research, Frederick, MD, USA.; ^6^ Laboratory of Cancer Biology and Genetics, National Cancer Institute, National Institutes of Health, Bethesda, MD, USA.; ^7^ Genome Modification Core, Laboratory Animal Sciences Program, Frederick National Laboratory for Cancer Research (FNLCR), Frederick, MD, USA.; ^8^ Protein Mass Spectrometry Group, Center for Cancer Research, National Cancer Institute, National Institutes of Health, Frederick, MD, USA.; ^9^ CCR Collaborative Bioinformatics Resource (CCBR), Frederick National Laboratory for Cancer Research, Leidos Biomedical Research Inc., Frederick, MD, USA.; ^10^ Molecular Histopathology Laboratory, Laboratory Animal Sciences Program, Frederick National Laboratory for Cancer Research, Frederick, MD, USA.

## Abstract

Transfer RNA (tRNA) modifications are crucial for protein synthesis, but their position-specific physiological roles remain poorly understood. Here, we investigate the impact of *N*^4^-acetylcytidine (ac^4^C), a highly conserved tRNA modification catalyzed by the essential acetyltransferase Nat10. By targeting Thumpd1, a nonessential adapter protein required for Nat10-catalyzed tRNA acetylation, we determine that loss of tRNA acetylation leads to reduced levels of tRNA^Leu^, increased ribosome stalling, and activation of eIF2α phosphorylation. Thumpd1 knockout mice exhibit growth defects and sterility. Concurrent knockout of Thumpd1 and the stress-sensing kinase Gcn2 causes penetrant postnatal lethality in mice, indicating a critical genetic interaction. Our findings demonstrate that a modification restricted to a single position within type II cytosolic tRNAs can regulate ribosome-mediated stress signaling in mammalian organisms, with implications for our understanding of translational control and therapeutic interventions.

## Teaser

An ancient tRNA modification is used by mammalian cells to coordinate protein translation and adaptive signaling.

## INTRODUCTION

Transfer RNAs (tRNAs) are fundamental to protein synthesis, serving as the physical connection between amino acids and the nucleotide triplet genetic code. A conspicuous trait of these molecules observed across all organisms is their decoration by >100 structurally diverse RNA modifications (*1*–*3*). Metazoan tRNAs contain, on average, ~13 modified nucleotides per molecule. However, these modifications are not distributed evenly, with some reaching near ubiquity and others being quite rare, found on only a small subset of tRNA isoacceptors. Prominent among this latter more restricted class is RNA cytidine acetylation.

**Fig. 1.**
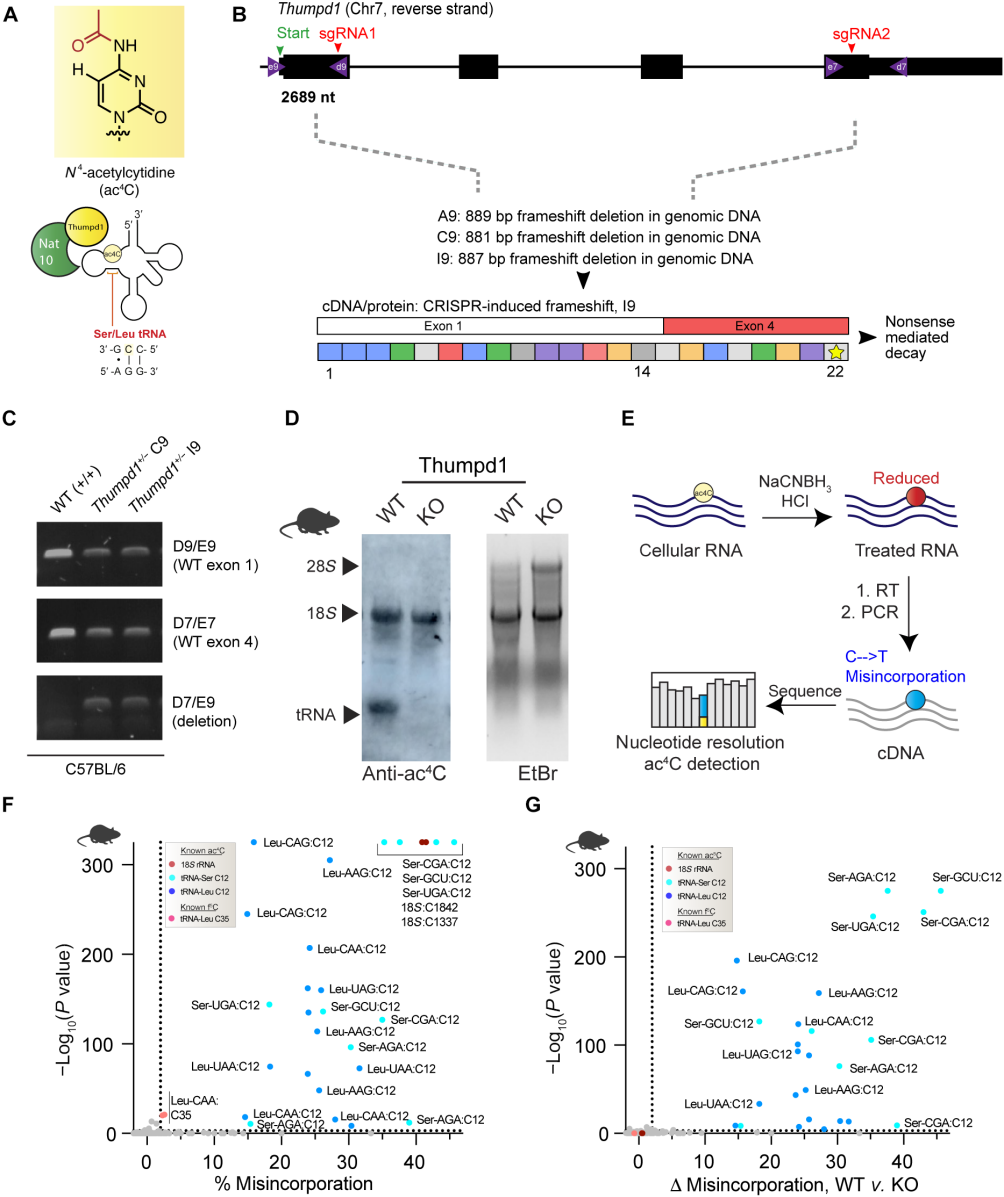
In vivo model for studying mammalian tRNA acetylation. (**A**) Deposition of the RNA modification ac^4^C in eukaryotic tRNA requires Nat10 and Thumpd1. (**B**) Schematic of murine *Thumpd1* locus and CRISPR-Cas9 genome editing strategy. Multiple murine lines (A9, C9, and I9) were obtained, and genotypes were characterized by next-generation sequencing. (**C**) PCR-based genotyping confirms *Thumpd1* deletion. (**D**) Immuno-Northern blotting confirms loss of ac^4^C in tRNA upon Thumpd1 KO. EtBr, ethidium bromide. (**E**) Schematic for ac^4^C-seq. (**F**) Distribution of ac^4^C in unfractionated murine tRNA-enriched total RNA. Data represent mean values from three independent mouse liver samples (*n* = 3 biological replicates). (**G**) Applying ac^4^C-seq to unfractionated total RNA isolated from WT or Thumpd1 KO mice highlights C12 in the D-arm of tRNA^Leu/Ser^ as the dominant position of Thumpd1-regulated RNA acetylation. Data represent mean values from three independent mouse liver samples (*n* = 3 biological replicates per genotype).

*N*^4^-acetylcytidine (ac^4^C) is a highly conserved RNA modification found across all domains of life (Fig. 1A) (*4*). In humans and mice, ac^4^C is introduced into RNA posttranscriptionally by the essential acetyltransferase *N*-acetyltransferase 10 (NAT10) (*5*, *6*). One unique property of this enzyme is its ability to modify both tRNA and ribosomal RNA (rRNA). To address these distinct substrates, Nat10 uses specific adapters, which, for tRNA, include THUMP domain-containing protein 1 (Thumpd1). The role of THUMP domain proteins in ac^4^C biogenesis was first discovered in *Saccharomyces cerevisiae*, where genetic screens that found deletion of a Thumpd1 homolog (Tan1) causes synthetic lethality in strains harboring a mutated allele of the single copy tRNA^Ser^_CGA_ gene (*7*, *8*). Thumpd1 was found to be required for formation of ac^4^C at position C12 in the D-arm of the tRNA^Ser^ and tRNA^Leu^. Subsequent studies found that loss of ac^4^C reduced the levels of these type II tRNAs in *S. cerevisiae* (tRNA^Ser^) (*9*) and, more recently, *S. pombe* (tRNA^Leu^) (*10*) and can cause growth defects at higher temperatures, consistent with its biophysically stabilizing role (*11*). Disruption of tRNA ac^4^C in yeast can also activate the general amino acid control pathway (*10*), suggestive of a connection to ribosome-mediated activation of Gcn2 (*12*–*14*). In humans, rare biallelic mutations in *THUMPD1* are associated with loss of ac^4^C in tRNA and neurodevelopmental defects (*15*).

A paradox in RNA modification biology is that, even as the number of putative RNA modification sites known has rapidly increased, analyses of individual positions and their physiological impacts remain rare (*3*). In models like yeast RNA modification, mutants often exhibit subtle phenotypes, motivating their dissection in multicellular organisms, where more pronounced and diverse phenotypes have the opportunity to present themselves (*16*–*18*). Toward this goal, here, we investigate the physiological effects of tRNA ac^4^C in a mouse model of Thumpd1 loss. Using ac^4^C-seq, we find that across evolution, the dominant sites of tRNA acetylation are tightly confined to the D-arm of tRNA^Ser^ and tRNA^Leu^ isodecoders. Thumpd1 knockout (KO) mice, which lack tRNA acetylation, are viable but show sub-Mendelian birth ratios, exhibit growth defects, and fail to reproduce. Characterizing a human THUMPD1 KO cell line yields evidence that loss of tRNA acetylation is associated with reduced levels of tRNA^Leu^, ribosome stalling, and ribosome collisions. This stimulates phosphorylation of the translation factor eIF2α, causing a general defect in protein synthesis. Similar evidence for reduced tRNA^Leu^ and eIF2α phosphorylation is observed in cerebella isolated from Thumpd1 KO mice. Last, we show that dual KO of Thumpd1 and Gcn2 causes penetrant postnatal lethality, linking tRNA acetylation to eIF2α kinase activity in vivo. Overall, our findings are consistent with the ability of a restricted and nonessential tRNA modification to regulate signaling through the ribosome in a multicellular organism, with implications for human physiology and disease.

## RESULTS

### tRNA acetylation can be manipulated in vivo via Thumpd1

To develop an in vivo model for studying mammalian tRNA acetylation, we constructed a heterozygous KO mouse (*Thumpd1^+/−^*) by targeting sites in exons 1 and 4 of the *Thumpd1* gene located on chromosome 7 using CRISPR-Cas9. Next-generation sequencing of the *Thumpd1* locus revealed the formation of large [~880 base pair (bp)] frameshift deletions in several lines, which could be readily differentiated from the wild-type (WT) allele by polymerase chain reaction (PCR) (Fig. 1, B and C). To measure the expression of Thumpd1, we analyzed Thumpd1 mRNA levels in the liver isolated from WT, heterozygous (*Thumpd1^+/−^*), and KO (*Thumpd1*^−/−^) variants. Thumpd1 transcript levels directly correlated with the presence of an intact allele, with WT mice expressing the highest and KO mice the lowest amount (fig. S1A). Anti-ac^4^C immunoblotting confirmed loss of tRNA acetylation (lower band) but not rRNA acetylation (top band) in the KO line (Fig. 1D).

Next, we sought to better understand the spectrum of tRNAs marked by Thumpd1-dependent cytidine acetylation. In our previous studies—as well as those of others—ac^4^C has been identified exclusively at C12 within the D-arm of eukaryotic tRNA^Leu/Ser^ (*7*, *19*). However, it is not known whether ac^4^C is a pervasive characteristic of tRNA^Leu/Ser^ or marks only a subset of isoacceptors and/or isodecoders. Furthermore, a few recent studies have suggested a broader role for ac^4^C within tRNAs (*20*–*22*). To characterize this, we determined the presence of cytidine acetylation in eukaryotic tRNAs using ac^4^C sequencing (ac^4^C-seq) (Fig. 1E). This nucleotide resolution sequencing reaction uses acidic NaCNBH_3_ to alter the structure of ac^4^C, resulting in misincorporations at modification sites upon reverse transcription (RT) (*23*). These can then be identified as mutations by cDNA sequencing. The value of ac^4^C-seq in discovery applications has been validated across multiple settings (*19*, *24*). Pooling three replicate ac^4^C-seq analyses of tRNA-enriched total RNA isolated from livers of WT and Thumpd1 KO mice, we sampled over 9603 tRNA positions at depths of >100 reads, including over 75% of known tRNA^Ser^ and tRNA^Leu^ genes. NaCNBH_3_-dependent misincorporations were observed across all tRNA^Ser^ and tRNA^Leu^ genes sampled, which summed 26 in total and included all nine isoacceptors (Fig. 1F and data S1A). In all cases, modifications occurred at C12. Misincorporation signals were, overall, more penetrant for tRNA^Ser^ than tRNA^Leu^. However, careful inspection of the sequence coverage identified a higher propensity for stops adjacent to C12 of tRNA^Leu^ (fig. S1B). This is an important illustration that, although ac^4^C-seq is quantitative for individual sites, it is not absolute; misincorporation signals across sites may vary due to both reduced ac^4^C stoichiometry and each individual RNA’s propensity for stop versus readthrough. Strong misincorporation signals were also observed at C35 of tRNA^Leu^_CAA_, a known 5-formylcytidine (f^5^C) site. We have previously defined the cross-reactivity of f^5^C with the ac^4^C-seq chemistry (*25*). Other sites producing significant C > T signals upon acidic NaCNBH_3_ treatment were characterized by low (<2%) misincorporation ratios. In addition to their low stoichiometry, these sites did not occur at the 5′-CCG-3′ consensus sequence whose modification Nat10 is known to favor and were not sensitive to Thumpd1 KO (Fig. 1G and data S1B). To see whether we could identify additional penetrant cytidine acetylation sites in tRNA, we used ac^4^C-seq to analyze small RNA fractions isolated from additional yeast, mouse, and human models. Here again, we found modification sites conserved across models to be confined to tRNA^Leu/Ser^ C12 (fig. S1, C to E, and data S1C). We further corroborated that C12 tRNA^Ser^_CGA_ acetylation is abolished in Thumpd1 KO mice using amplicon sequencing (fig. S1F). Taken as a whole, our results indicate that the most penetrant sites of tRNA acetylation are confined to the D-arm of tRNA^Ser^ and tRNA^Leu^ across eukaryotes, occur across all detected isoacceptors, and are highly dependent on the presence of Thumpd1.

### Thumpd1 loss in mice is associated with small size and sterility

**Fig. 2.**
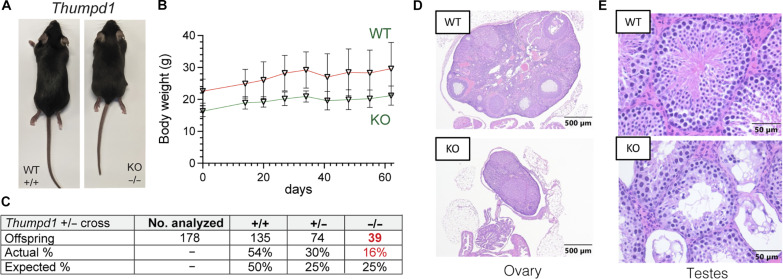
In vivo phenotypes associated with loss of Thumpd1 and tRNA acetylation. (**A**) *Thumpd1^−/−^* mice are runted. (**B**) *Thumpd1^−/−^* mice are viable for several weeks. (**C**) Offspring produced in *Thumpd1^+/−^* breeding studies. (**D**) Ovarian atrophy and (**E**) testicular seminiferous tubule degeneration in *Thumpd1^−/−^* mice. Immunohistochemistry results are representative of *n* = 4 biological replicates per genotype.

Heterozygous *Thumpd1*^+/−^ mice are viable and fertile and do not present any overt phenotype. However, mice completely deficient in Thumpd1 (*Thumpd1^−/−^*) have significantly smaller body sizes, suggesting an important role for this gene in growth (Fig. 2A). Despite their small size, adult *Thumpd1^−/−^* mice are viable as assessed by maintenance of body weight over time (Fig. 2B). Breeding studies found that *Thumpd1^−/−^* mice are born at sub-Mendelian ratios indicative of decreased pre- or perinatal fitness (Fig. 2C). Ovarian atrophy in KO mice is characterized histologically by decreased ovarian oocytes, developing follicles, and corpora lutea and replacement with interstitial cell hyperplasia (Fig. 2D). Testicular atrophy is characterized histologically by disorganized seminiferous tubules with loss or vacuolation of germ cells and multinucleated germ cells; exfoliated germ cells are also observed within the epididymis (Fig. 2E). Terminal deoxynucleotidyl transferase–mediated deoxyuridine triphosphate nick end labeling (TUNEL) staining was significantly increased in testes but not the liver, aligning with the selective pathology (fig. S2). Further consistent with these observations, repeated matings of male *Thumpd1^−/−^* mice (6 to 21 weeks of age) with WT females and female *Thumpd1^−/−^* mice (6 to 21 weeks of age) with WT males failed to produce pregnancies. In contrast to the neurodevelopmental defects observed in human patients (*15*), no obvious changes in brain weight or morphology were observed in KO mice. These findings define overt phenotypes that accompany loss of Thumpd1-regulated tRNA acetylation in an animal model.

### THUMPD1 regulates overall levels and ribosome occupancy of tRNA^Leu^

**Fig. 3.**
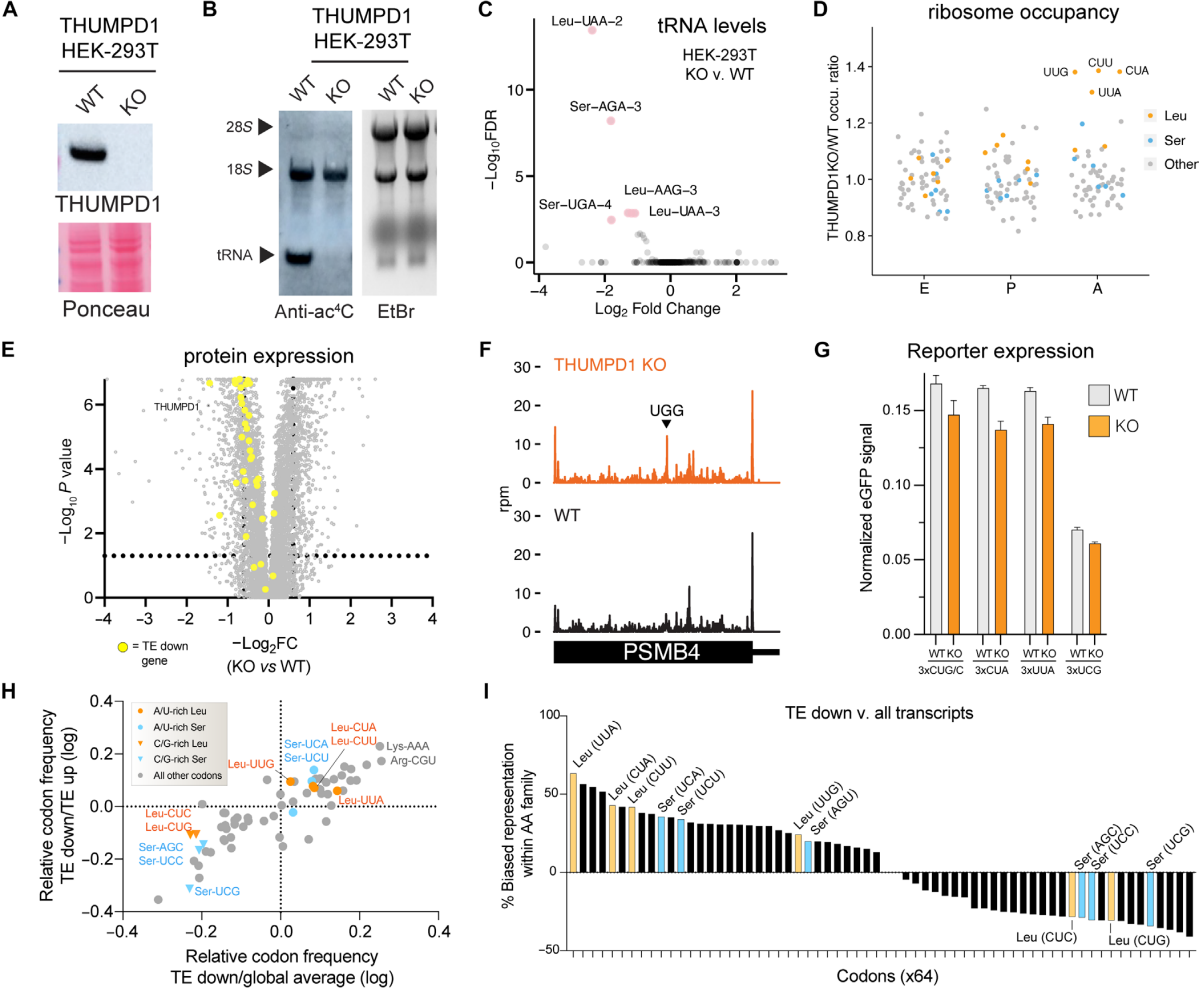
Molecular characterization of THUMPD1/tRNA acetylation in mammalian cells. (**A**) Western blot confirms THUMPD1 KO. Data are representative of *n* = 3 biological replicates. (**B**) Immuno-Northern blotting confirms loss of tRNA ac^4^C upon THUMPD1 KO (HEK-293T). Data are representative of *n* = 2 biological replicates. (**C**) mim-tRNA-seq analysis of WT v. THUMPD1 KO cells indicates the effect on tRNA^Leu/Ser^ isodecoders. Data are derived from *n* = 2 biological replicates. (**D**) Ribosome profiling of WT v. THUMPD1 KO cells indicates ribosome pausing at codons (UUG, CUU, CUA, and UUA) decoded by tRNA^Leu^ in the ribosomal A-site. Codons encoding Leu, Ser, and other amino acids are shown in blue, orange, and gray, respectively. Data are derived from *n* = 4 biological replicates. (**E**) Proteomic profiling of WT v. THUMPD1 KO cells. Genes showing decreased TE (TE down) are highlighted in yellow. Values derived from *n* = 3 biological replicates. (**F**) Ribosome footprints in PSMB4 gene indicates that stalling at Leu codons in KO cells does not limit downstream ribosome occupancy. (**G**) eGFP reporters with 3x copies of Leu or Ser codons do not produce significantly less protein in THUMPD1 KO cells. Data are derived from *n* = 3 technical replicates. (**H**) Scatterplot of codon frequency in mRNAs differentially translated in Thumpd1 KO cells. Global average refers to average codon usage of all CCDS-defined consensus coding sequences. Values shifted up/right indicate codons more frequent in TE down transcripts. Leu codons: orange; Ser codons: blue. (**I**) Analysis of amino acid family–specific codon bias in TE down transcripts. Representation of individual codons (e.g., Leu-UUA) relative to amino acid family (e.g., all Leu) was calculated. Average values for TE down sequences and all CCDS-defined coding sequences were compared. Leu codons; orange; Ser codons: blue. U/A-rich Leu codons are labeled on the *x* axis in red.

As a model system for mechanistic studies, we used CRISPR-Cas9 to delete THUMPD1 in an immortalized human embryonic kidney (HEK) 293T cell line. Our rationale was that working in human cells might highlight commonalities shared across eukaryotes while, moreover, providing a model technically tractable to analysis by a wide range of experimental techniques. Similar to observations in mice, targeting of the *THUMPD1* gene using CRISPR-Cas9 resulted in loss of THUMPD1 protein and specific abrogation of tRNA acetylation (Fig. 3, A and B). Previous studies have found that deletion of the *S. cerevisiae* Thumpd1 homolog decreased levels of ac^4^C-containing tRNAs (*9*, *26*). However, Northern blotting analysis did not detect clear changes in tRNA^Leu/Ser^ levels THUMPD1 KO cells (fig. S3), consistent with prior results in a HeLa cell line (*15*). To increase the sensitivity of our analysis, we performed unbiased profiling of tRNAs in our WT and KO models using modification-induced misincorporation tRNA sequencing (mim-tRNA-seq) (*27*). A strength of this method is its ability to assay levels of sequence-related tRNA isodecoders that are difficult to differentiate by hybridization. This revealed that THUMPD1 KO specifically affects the levels of ac^4^C-containing tRNAs in HEK-293T cells, causing an ~4-fold decrease in levels of tRNA^Leu^_UAA-2_ and smaller reductions in tRNA^Ser^_AGA-3_, tRNA^Ser^_UGA-4_, tRNA^Leu^_AAG-3_, and tRNA^Leu^_UAA-3_ (Fig. 3C and data S2). To define whether these subtle perturbations affected translation, we performed ribosome profiling in THUMPD1 WT and KO cells. Our data showed that loss of THUMPD1 results in increased A-site occupancy at UUG, CUA, CUU, and UUA codons (Fig. 3D and data S3). Invoking wobble rules, all four of these can be decoded by tRNA^Leu^_UAA_ and tRNA^Leu^_AAG_ (more often found as tRNA^Leu^_IAG_) (*28*). Both of these species are detected as down-regulated in our mim-tRNA-seq data. To better understand why stalling is selective for Leu but not Ser codons, we aggregated our mim-tRNA-seq data and evaluated total tRNA abundance at the anticodon level, summing all isodecoders that decode each codon. This revealed significant reductions in multiple tRNA^Leu^ populations, whereas tRNA^Ser^ remained largely unchanged (fig. S4). Our data contrasts with that of Darnell and co-workers, who found ribosomes were recalcitrant to stalling at Leu codons even upon leucine deprivation (*29*) and better matches the observation of Chou and co-workers, who detected altered ribosome occupancy at Leu codons upon deletion of the *S. cerevisiae* Thumpd1 homolog (*30*). Global proteomics and immunoblotting indicated that genes with decreased translation efficiency (TE) in THUMPD1 KO cells were modestly down-regulated at the protein level (Fig. 3E and data S4). Surveys of transcript level data for mRNAs such as PSMB4 (Fig. 3F) and CDK1 (fig. S5) indicate that, even in transcripts where strong pauses at tRNA^Leu^-decoded codons can be visualized, ribosome occupancy is still observed downstream. This suggests that, even when THUMPD1-dependent pausing occurs at Leu codons, protein production may be unburdened.

One way tRNA modifications can shape the proteome is by influencing codon-biased translation (*31*–*36*). To test whether a similar phenomenon acted in THUMPD1 KO cells, we performed comparisons using a reporter containing multiple copies of Leu codons that exhibited stalling (UUA and CUA) or that did not exhibit stalling (CUG/C and UCG) upstream of an enhanced green fluorescent protein (eGFP) gene. No significant difference in protein production was observed relative to WT cells (Fig. 3G). To explore this phenomenon further, we analyzed codon composition in genes showing altered TE in THUMPD1 KO cells (data S5). Comparing canonical transcripts found in the TE up and TE down datasets (data S5), we found the latter contained a significantly greater percentage of the U/A-rich Leu codons that showed stalling in our ribosome profiling studies (UUG, CUA, CUU, and UUA) relative to C/G-rich Leu codons that did not show stalling (CUC and CUG; Fig. 3H, *y* axis, and fig. S6A). Compared to codon composition of all human genes, U/A-rich Leu codons were also modestly enriched (Fig. 3H, *x* axis, and fig. S6, B and C). Analysis of amino acid pairs within TE down transcripts also found modest enrichment of diamino acids containing U/A-rich Leu (table S5) (*37*). Evaluating the skew between representations of individual codons (e.g., Leu-UUA) relative to the entirety of their amino acid family (e.g., all Leu) in TE down coding sequences revealed a clear bifurcation between U/A- and C/G-rich Leu/Ser (Fig. 3I). The former are more enriched TE down mRNAs, offering additional evidence of U/A-rich Leu/Ser bias. Whereas TE down sequences exhibit a shift in Leu codon composition favoring U/A-rich variants, other codons such as Lys-AAA and Arg-CU exhibit the highest absolute frequencies of enrichment (Fig. 3H). This could indicate that context, rather than frequency, determines the effect of Thumpd1 KO on protein translation. As one example, association of positively charged amino acids with the ribosomal exit tunnel can decrease translational velocity (*38*) and cause premature termination (*39*, *40*), raising the possibility that THUMPD1 may collaborate with this or other transcript features. Alternatively, the apparent bias of arginine and lysine codons may reflect a pathway effect as many TE-affected genes are ribosomal proteins and translation factors known to be enriched in positively charged amino acids (*41*). Our results paint a picture that requires holding two opposing ideas in mind: First, sequence biases exist that imply—but do not demonstrate—a subtle, context-dependent impact of THUMPD1 on codon-specific translation. Second, the magnitude of this effect appears insufficient to disrupt reporter gene expression or obviously account for the extensive remodeling of HEK-293T proteomes caused by loss of tRNA ac^4^C.

### THUMPD1 loss stimulates phosphorylation of eIF2α

**Fig. 4.**
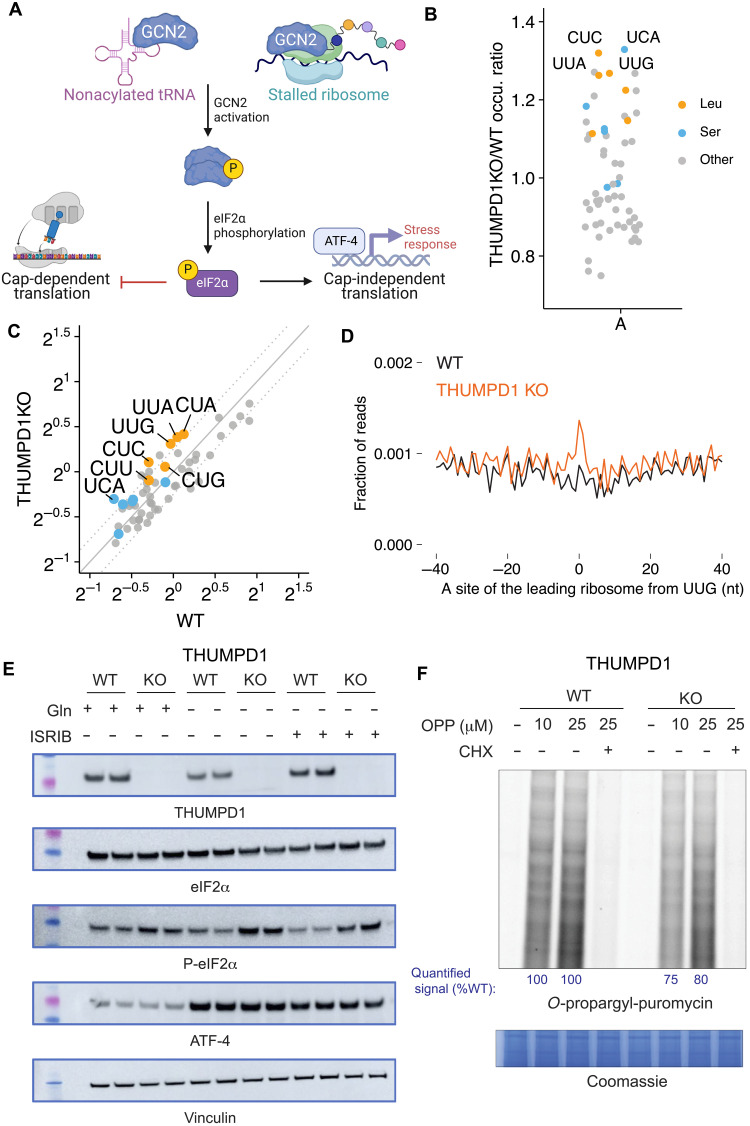
THUMPD1-mediated tRNA acetylation regulates ribosome collisions. (**A**) Defective tRNA function can activate the sensor kinase GCN2 and trigger the ISR. (**B**) Disome occupancies of WT v. THUMPD1 KO cells at the ribosomal A-site of the leading ribosome indicate increased disome formation at codons (UCA, CUC, UUG, and UUA) decoded by tRNA^Leu/Ser^. Codons encoding Ser, Leu, and other amino acids are shown in orange, blue, and gray, respectively. Data are representative of *n* = 2 biological replicates. (**C**) Scatterplot of disome occupancies at 61 sense codons in THUMPD1 KO v. WT cells indicate increased ribosome collisions at multiple codons decoded by tRNA^Leu/Ser^. (**D**) Meta-codon plot centered at UUG codons shows increased ribosome collisions in THUMPD1 KO (orange) compared to WT cells (black). (**E**) Analysis of eIF2a phosphorylation in THUMPD1 KO cells. Amino acid deprivation (−Gln) and ISRIB (1 μM) were used to induce translational stress. Biological replicates are loaded in adjacent lanes. (**F**) Analysis of global translation in THUMPD1 KO cells. OPP was used to label nascent transcripts, which were then ligated to a fluorophore-azide and visualized via SDS-PAGE. Densitometry analysis was calculated using the ImageJ software and is graphed in fig. S7I. CHX, cycloheximide.

As an alternative to codon-specific effects, we considered what signaling pathways may help cells maintain homeostasis in response to reduced tRNA^Leu/Ser^ levels. The integrated stress response (ISR) is major cellular mechanism used to adapt to changing environmental conditions including nutrient deprivation, pathogens, heme deficiency, and endoplasmic reticulum (ER) stress (*42*). Uncharged tRNAs and/or ribosomal collisions activate the sensor kinase Gcn2, which, in turn, phosphorylates the translation factor eIF2α at Ser^51^ (Fig. 4A). This phosphorylation event promotes formation of a tight eIF2α/eIF2β complex incapable of the nucleotide recycling necessary for canonical cap-dependent translation. Instead, proteins are produced primarily from mRNAs whose translation is recalcitrant to this event such as the stress-responsive transcription factor ATF4. As a previous study has found uncharged tRNAs to be more rapidly degraded than their charged counterparts (*43*), we hypothesized that the lower levels of tRNA^Ser^ and tRNA^Leu^ in THUMPD1 KO cells may reflect reduced aminoacylation. However, applying both RT quantitative PCR (RT-qPCR) and hybridization-based assays, we did not observe significant evidence for decreased charging of tRNA^Leu^ in THUMPD1 KO cells (fig. S7, A and B). This is consistent with a recent study that found that deletion of the yeast Thumpd1 homolog does not affect tRNA charging (*44*). To probe for evidence of ribosome collisions, we applied disome profiling to profile mRNA fragments differentially protected by endogenous stacked ribosomes in the WT and THUMPD1 KO cell lines. High-resolution footprinting revealed increased A-site occupancy across several codons in THUMPD1 KO cells (Fig. 4, B and D, and table S6). Although amino acid–selective occupancy changes were not as pronounced as with monosome analyses, the greatest differences were observed at Ser (UCA) and Leu (UUG, UUA, and CUC; Fig. 4, B and D) codons. Each of these is decoded by an ac^4^C-containing tRNA.

To determine whether ribosome collisions were associated with increased ISR kinase activation, we analyzed lysates derived from THUMPD1 KO cells with a site-specific Ser^51^ P-eIF2α antibody. A subtle but reproducible increase in P-eIF2α was observed in THUMPD1 KO cells (Fig. 4E), which became more apparent upon glutamine (Gln) deprivation or integrated stress response inhibitor (ISRIB) treatment. Phosphorylation of eIF2α was also apparent in the previously reported THUMPD1 KO HeLa cell line (fig. S7C) (*15*) and, in both cell lines, was sensitive to treatment with a Gcn2 inhibitor (fig. S7, D and E). THUMPD1 KO was associated with a growth defect in HeLa but not HEK-293T (fig. S7F). In both HEK-293T and HeLa cells, THUMPD1 KO by itself was not sufficient to activate ATF4 (Fig. 4E and fig. S7C). This is in line with our proteomic and transcriptomic data, which did not find up-regulation of canonical ATF4 targets such as asparagine synthetase (ASNS) in THUMPD1 KO cells (fig. S7G). A genome-wide screen for previously unidentified regulators of ATF4 activation also did not identify THUMPD1 (*45*). The threshold at which Gln deprivation activates ATF4 expression appeared similar in WT and THUMPD1 KO HEK-293T cells (fig. S7H). This suggests either THUMPD1 is not a sufficiently strong stimulus to activate ATF4 or that our cell lines harbor compensatory mechanisms that blunt ISR activation in response to permanent THUMPD1 loss.

As increased levels of P-eIF2α would be expected to impede translation, we assessed nascent protein synthesis by subjecting WT and KO cells to labeling by *O*-propargyl puromycin (OPP). Loss of THUMPD1 was associated with significantly lower levels of OPP incorporation, consistent with a translation defect (Fig. 4F and fig. S7, I to L). In addition to reduced translation caused by eIF2α phosphorylation, our proteomic data also observed a significant decrease in ribosomal proteins in THUMPD1 KO cells (fig. S7G and data S12). This is in line with the notion that serial passage of THUMPD1 KO cells may select for clones that adapt to increased ribosome collisions by down-regulating the translational machinery. Consistent with this view, genome-wide Perturb-seq recently found that the single-cell RNA sequencing (RNA-seq) signature of THUMPD1 KO was most similar to those elicited by disrupting of genes involved in ribosome biosynthesis, including POLR1A, POLR1B, POLR1C, and POLR1E (fig. S7M) (*46*). Examination of THUMPD1 KO cells did not reveal changes in mTOR activation, suggesting that the effects of THUMPD1on ribosomal protein levels are not mediated by this pathway (fig. S7N). Because of the aforementioned potential for compensatory changes to be selected for in KO cell lines, care must be taken not to overinterpret these findings. However, the identification of a link between THUMPD1, eIF2α phosphorylation, and translational down-regulation is consistent with recent studies in model organisms (*10*, *47*). These results indicate that loss of THUMPD1-dependent ac^4^C can cause ribosome collisions associated with eIF2α phosphorylation and defective translation, spurring us to explore the significance of this mechanism in vivo.

### Thumpd1 genetically interacts with Gcn2 in vivo

**Fig. 5.**
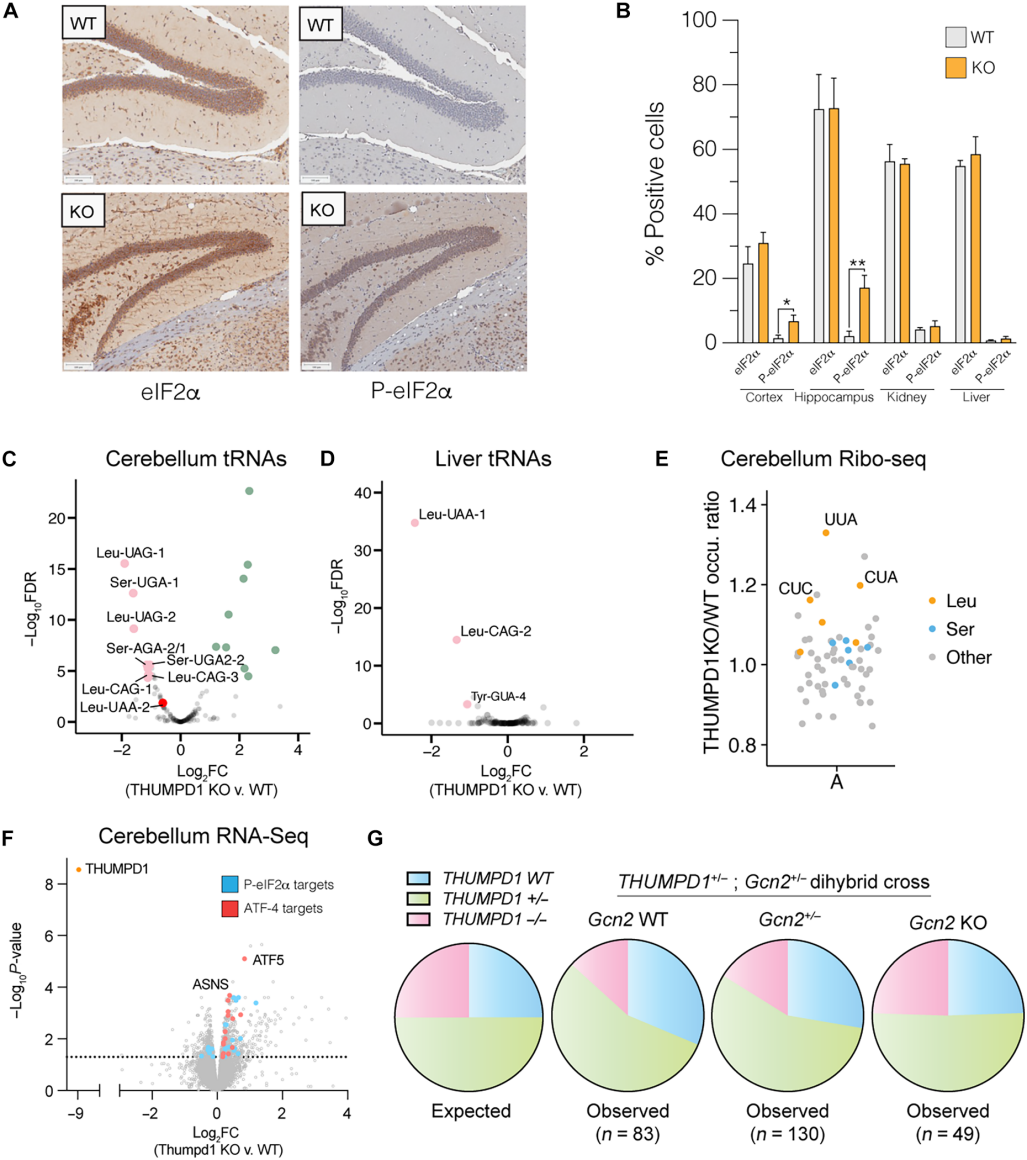
Thumpd1 and Gcn2 interact in vivo. (**A**) IHC staining of total eIF2a (left) and (Ser^51^) P-eIF2a (right) in brain tissue isolated from age-matched WT and Thumpd1 KO mice. Data are representative of *n* = 4 biological replicates. (**B**) Quantification of percent positive cells in the cerebral cortex, hippocampus, kidney cortex, and liver. (**C** and **D**) mim-tRNA-seq analysis of tRNA levels in the cerebellum (C) and liver (D) of WT and Thumpd1 KO mice shows reduced levels of tRNA^Leu/Ser^ isodecoders. Green points represent nonacetylated tRNA species that showed significant increases in abundance not marked by ac^4^C. Data are derived from *n* = 2 biological replicates for each tissue. (**E**) A-site ribosome occupancies of cerebella isolated from WT and THUMPD1 KO mice indicate increased ribosome stalling at codons (UUA, CUA, and CUC) decoded by tRNA^Leu^. Codons encoding Leu, Ser, and other amino acids are shown in orange, blue, and gray, respectively. Data are representative of *n* = 2 biological replicates. (**F**) Gene expression profiling of cerebella isolated from WT and Thumpd1 KO mice. Literature annotated targets of Atf4 (red) and P-eIF2a (blue) exhibit a skew toward greater expression upon Thumpd1 KO. Values represent the average of *n* = 4 biological replicates. (**G**) Analysis of offspring produced by *Thumpd1^+/−^*, *Gcn2^+/−^* dihybrid cross. Pie charts are organized by *Gcn2* genotype, with WT on the left, heterozygotes in middle, and KO on the right. The proportion of mice born on a given background is color coded as follows: blue: *Thumpd1^+/+^*; green: *Thumpd1^+/−^*; pink: *Thumpd1^−/−^*. Full numbers for dihybrid cross are provided in fig. S11A.

Next, we sought to understand whether our observations from HEK-293T cells were relevant in vivo. As an initial test, we isolated tissues from age-matched WT and Thumpd1 KO mice and assessed levels of P-eIF2α by immunohistochemical (IHC) staining (Fig. 5A). Whereas eIF2α levels were statistically identical across all samples, the percentage of P-eIF2α positive cells was significantly increased in cryosections isolated from the brain (hippocampus and cerebral cortex; Fig. 5B). This change was organ specific as P-eIF2α levels in the kidney and liver were not affected (Fig. 5B and fig. S8). Applying mim-tRNA-seq to small RNA fractions isolated from the cerebellum and liver, we again observed tRNA^Leu^ and tRNA^Ser^ to be the most down-regulated (Fig. 5, C and D, and data S7). Consistent with the increased eIF2α phosphorylation observed in brain tissues, more isodecoders were affected in the cerebellum (*7*) than liver (*2*). Cerebella also displayed up-regulation of several tRNA isodecoders (Fig. 5C, green). As these tRNAs do not contain Thumpd1-dependent ac^4^C and did not change in other tissues, it is possible that their alteration may represent technical noise or secondary effects. Ribosome profiling of cerebellum tissue samples indicated that loss of Thumpd1 causes the greatest increase in occupancy at the Leu codon UUA, consistent with the potential for loss of tRNA acetylation to stimulate ribosome collisions and eIF2α phosphorylation (Fig. 5E and data S8).

To further investigate the consequences of Thumpd1 KO in the cerebellum, we carried out RNA-seq analysis. A recent study found that exit from pluripotency is associated with eIF2α phosphorylation and used WT and eIF2α S51A mouse embryonic stem cells to define a set of transcripts dependent on eIF2α phosphorylation upon withdrawal of “2i” [two chemical inhibitors of MEK1/2 (mitogen-activated protein kinase kinase 1/2) and GSK3α/β (glycogen synthase kinase 3α/β)] (*48*). Overlaying these genes onto our dataset, we found most of them were up-regulated upon Thumpd1 KO, consistent with eIF2α phosphorylation (Fig. 5F, cyan, and data S9). To assess activation of the ISR, we further compared our differentially expressed gene set to Atf4 target genes previously identified in mouse embryonic fibroblasts (Fig. 5F, red) (*12*, *49*). Here, we found a statistically significant up-regulation of several ISR genes including Atf4 itself (FC_KO v. WT_ = 1.25x, adjusted *P* value = 8.7 × 10^−4^). Evaluation of ribosome occupancy across the Atf4 transcript also revealed increased ribosome density in THUMPD1 KO samples, consistent with enhanced translation through relief of upstream open reading frame (uORF) repression (*50*), the canonical mechanism by which eIF2α phosphorylation promotes ATF4 expression (fig. S9). However, most eIF2α- and Atf4-dependent gene sets were unaffected, consistent with a modest response. Transcriptomic analysis of liver tissue did not show this signature (data S10). Notably, among many affected pathways, gene set enrichment analysis (GSEA) indicated up-regulation of inflammatory signaling in the cerebellum but down-regulation of the mTOR signaling in the liver, possibly indicating a selective compensatory response in the latter tissue (fig. S10, A and B, and tables S9 and S10). RNA-seq of testes, a tissue that exhibits obvious pathology upon Thumpd1 KO, provided additional evidence for tissue-selective impact with up-regulation of inflammatory and apoptotic gene expression pathways (fig. S10C and table S11). This correlated with reduced levels of tRNA^Leu^, although similar to cerebella, we observed that mim-tRNA-seq data was noisier relative to cell lines or tissues possibly reflecting more widespread dysfunction (fig. S10D). These findings indicate a degree of concordance between our in vitro and in vivo results and prompted us to explore links between Thumpd1 and the stress response in further detail.

Gcn2 is the most well-characterized sensor kinase involved in the tRNA-dependent ISR. In budding yeast (*S. cerevisiae*), deletion of a Gcn2 homolog protects from growth defects caused by the loss of tRNA body modifications such as ac^4^C, whereas in fission yeast (*S. pombe*), the opposite occurs (*9*, *10*, *26*, *47*). The Janus-like ability of Gcn2 to either propagate or mitigate tRNA-dependent phenotypes has also been observed in mammalian systems. Evidence indicates that an overactive ISR helps drive pathology in Charcot-Marie-Tooth (CMT) disease (*51*), a hereditary disorder that can be driven by dominant mutations in several tRNA synthetase genes. This has led to Gcn2 inhibition being explored as a therapeutic approach (*52*). Alternatively, the group of Ackerman has shown that activation of Gcn2 plays a protective role in animal models of cerebellar and retinal degeneration caused by dual mutations in the ribosome rescue factor GTPBP2 and central nervous system (CNS)–specific tRNA^Arg^ (*12*, *53*). However, the in vivo genetic interaction of Gcn2 with loss of a nonessential tRNA body modification has never been explored.

To test for a potential genetic interaction between *Thumpd1* and *Gcn2*, we generated and bred *Thumpd1*^+/−^, *Gcn2*^+/−^ males and females. *Gcn2*^−/−^ (*Thumpd1*^+/+^) mice fed a normal diet had no overt phenotype, consistent with a previous study (*54*). Genotyping of 262 offspring mice at day 14 (P14) after birth indicated that, on the *Gcn2^WT^* and *Gcn2^+/−^* backgrounds, ~13% of mice born were *Thumpd1^−/−^* (Fig. 5G and fig. S11A). The sub-Mendelian production of *Thumpd1^−/−^* mice is consistent with our initial breeding studies. However, of mice with a full KO (*Gcn2^−/−^*) background, ~22% of mice born were *Thumpd1^−/−^*. The increased frequency of *Thumpd1^−/−^* mice born on the *Gcn2^−/−^* background is statistically significant and suggests that suppression of Gcn2 activation in *Thumpd1^−/−^* mice beneficially affects prenatal fitness. In contrast, in the postnatal setting Gcn2 loss appeared to be extremely deleterious. *Gcn2^−/−^*, *Thumpd1^−/−^* mice were extremely runted (fig. S11, B and C) and uniformly perished before P30 even when extra care measures such as delayed weaning and heat pads were provided. The early lethality of *Gcn2^−/−^*, *Thumpd1^−/−^* dual KO made it challenging to identify and prepare these mice for histopathological analysis prior to decomposition. However, analysis of a limited number of animals (*n* = 4) found evidence for exacerbated phenotypes across multiple tissues, including one instance of cataract formation, increased germ cell degeneration, and a higher incidence of hydrocephalus, albeit with incomplete penetrance (fig. S11, D and E). The levels of cleaved caspase-3, an established marker of apoptosis, were significantly greater in double KO (DKO) tissues (fig. S12). Together, these results demonstrate the existence of a severe synthetic lethal genetic interaction between *Thumpd1* and *Gcn2* in mice.

## DISCUSSION

**Fig. 6.**
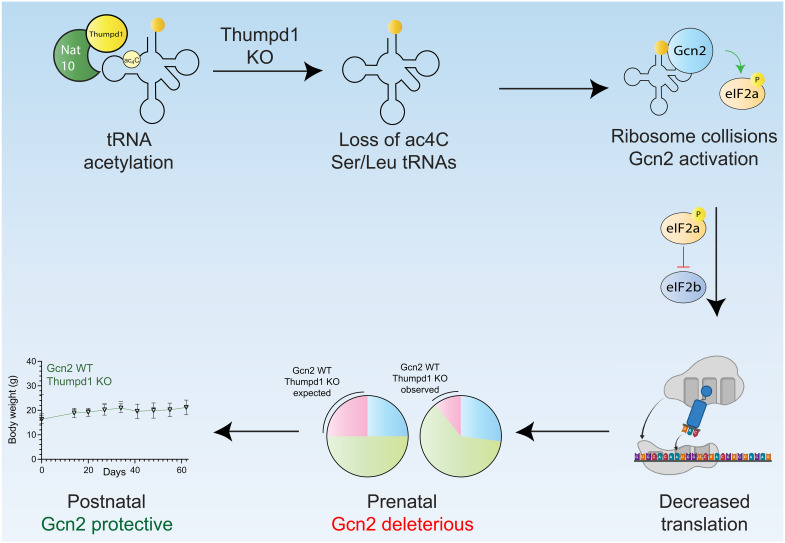
Model for Thumpd1-mediated tRNA ac^4^C in mammalian ribosome function. Nat10/Thumpd1 is responsible for C12 modification of tRNA^Leu^ and tRNA^Ser^. Genetic mutations and potentially other stimuli can cause a loss of ac^4^C from Ser/Leu tRNAs. This reduces the levels of tRNA^Leu/Ser^ isodecoders and possibly slows translocation, resulting in increased A-site occupancy at Leu/Ser codons and ribosome collisions. This activates the sensor kinase Gcn2, which causes tissue-specific phosphorylation of eIF2α. Cellular studies suggest that differential activation of Gcn2 may reflect the ability of tissues to access compensatory mechanisms upon ribosome collisions, for example, down-regulating ribosomal proteins. The sub-Mendelian production of *Thumpd1^−/−^* offspring is rescued by concurrent KO of *Gcn2*, suggesting that Thumpd1-dependent Gcn2 activation is deleterious during prenatal development. However, dual KO animals perish shortly after birth, suggesting that, in the postnatal setting, Gcn2 plays a protective role.

To date, the in vivo role of tRNA modification machinery has been most thoroughly characterized via enzymes targeting the anticodon and variable loops as well as mitochondrial tRNAs (*35*, *55*–*67*). Here, we harness in vivo disruption of a nonessential tRNA D-arm modification that specifically marks two cytosolic type II tRNAs to reveal a modification-dependent sensing mechanism that extends from yeast to vertebrates. Our research showcases how interrogating nonessential enzymes in vivo can uncover previously unidentified modification-driven phenotypes and proposes a model whereby Thumpd1-dependent tRNA acetylation influences cell fate based on the severity of translational stress its loss elicits (Fig. 6).

Our data suggest that tRNA acetylation can affect physiology by three distinct scenarios: homeostasis, adaptive response, and cellular dysfunction. In the homeostasis scenario, normal levels of tRNA acetylation maintain optimal tRNA levels and translational efficiency. The adaptive response regime is triggered when THUMPD1 loss leads to reduced tRNA acetylation, causing a decrease in tRNA^Leu^ and tRNA^Ser^ levels and increased ribosomal occupancy at their respective codons. Ribosome collisions activate the Gcn2-eIF2α axis, promoting a modest stress response that allows cells to adapt to the translational perturbation. In the cellular dysfunction regime, the lack of Gcn2—or tissue-specific compensatory factors that remain to be defined—enables translational stress to surpass a critical threshold, leading to apoptosis and organismal lethality.

Breeding studies revealed a complex interplay between Thumpd1 and Gcn2. Gcn2 loss appeared to improve prenatal fitness in Thumpd1-deficient mice, suggesting that excessive Gcn2 activation is detrimental to embryonic development. However, Thumpd1/Gcn2 DKO mice exhibited severe postnatal lethality, indicating a crucial protective role for Gcn2 in the postnatal setting. This finding also hints at other contexts where Thumpd1-mediated tRNA acetylation may be required. For instance, the parallels between embryonic development and cancer metastasis led us to carry out a pilot study in which heterozygous *Thumpd1^+/−^* mice—which harbor quantitatively lower levels of tRNA ac^4^C—were crossed into a model of breast cancer metastasis (fig. S13, A and B). Primary tumor burden was not affected in *Thumpd1^+/−^* mice; however, metastases were significantly repressed (fig. S13). Although we defer further characterization to future work, this experiment provides another example of the biological relevance of tRNA ac^4^C and the enabling nature of the models developed here.

Our work raises several questions for future investigation. First, the mechanism by which loss of ac^4^C triggered tRNA degradation remains undefined. Although the rapid decay of hypomodified tRNAs is well known in yeast (*9*, *68*), in mammals, these pathways are less extensively characterized. SLFN11 and SLFN12 are the most well-known regulators of type II tRNAs in mammals (*69*, *70*) but are not present in HEK-293T cells (*71*, *72*), raising the possibility of previously unidentified degradation mechanisms. Furthermore, we did not determine the precise cause of ribosome stalling in the absence of tRNA acetylation. In theory, A-site stalling could reflect rate-limited tRNA^Leu/Ser^ levels, altered activity of these tRNAs during translation, or some combination thereof. Previous studies have noted the proximity of the D-stem to helix 69 of rRNA during translocation (*44*, *73*), suggesting the potential for ac^4^C-dependent conformational dynamics to directly influence this process. In addition, tissue-specific adaptation to tRNA acetylation raises the question: Are there sensing mechanisms that directly link ac^4^C or tRNA^Leu/Ser^ to translational control? Our results provide a starting point for pursuing these questions, whose answers could afford critical insights into the role of tRNA modifications in development and disease.

Last, our studies suggest previously unidentified avenues that may be explored for treatment of THUMPD1-dependent neurodegenerative effects. Given that GCN2 KO potentiates the deleterious effects of THUMPD1 loss, we speculate that patients with THUMPD1 deficiency may benefit from strategies that increase eIF2α phosphorylation and reduce ribosome collisions. Genetic ablation of CHOP (*74*) and small molecule inhibition of the eIF2α phosphatase GADD34 (*75*) have shown promise in models of tRNA-related neurological disorders (*76*). In contrast, molecules such as ISRIB that activate eIF2α phosphorylation by amplifying ribosome collisions may be less desirable. A recent report described the ability of the serine/threonine kinase ZAKα (ZAK) to sense ribosome collisions and establish regimes of either tolerance or cell death in response to translational dysfunction (*77*). Although the role of this apoptotic mechanism in THUMPD1-dependent phenotypes is yet undetermined, clinically approved kinase inhibitors including nilotinib (BCR-ABL) and vemurafenib (BRAF) show crossover inhibition of ZAK, providing a potential approach to probe this mechanism. Another strategy to combat ribosome collisions would be to down-regulate the translational machinery, for instance, by mTOR inhibition. This strategy is supported by studies in yeast showing that ribosomal protein mutations can suppress growth defects caused by tRNA acetylation loss (*10*) and by our observations of compensatory ribosomal protein down-regulation in THUMPD1-deficient human cells. The in vivo models and phenotypes described in this study provide a robust foundation for testing these hypotheses. Collectively, our studies define the ability of tRNA acetylation to affect translational signaling in mammalian lineages, providing a conceptual foundation for understanding the role of this modification in biology and disease.

### Limitations of the study

Our work characterizes the occurrence of ac^4^C at C12 across a variety of tRNA^Ser^ and tRNA^Leu^ isodecoders. However, we cannot rule out that additional sites of tRNA acetylation may occur in different cell types or tissues. Similarly, our in vitro mechanistic studies were confined to a human cell line and do dismiss the possibility that more profound codon-biased translation may be regulated by THUMPD1-dependent ac^4^C in other settings. Delineating these and other potential mechanistic inputs will be important for a holistic understanding of how tRNA acetylation influences translation.

Because the only characterized function of THUMPD1 is to assist deposition of ac^4^C in eukaryotic tRNA, a parsimonious model is that the phenotypes observed in our study reflect changes in ac^4^C-dependent tRNA function. This notion is supported by the fact that ectopic expression of tRNA^Ser^ can rescue *S. cerevisiae* growth defects caused by mutation of a Thumpd1 homolog (*9*, *26*). However, we cannot rule out the possibility that THUMPD1-dependent phenotypes may stem from an uncharacterized chaperone role of this protein (*78*). Structural and biochemical analyses of the eukaryotic NAT10/ THUMPD1 complex will be important to constructing mutants that can more precisely modulate THUMPD1’s activity.

Our strategy for modulating ac^4^C contrasts with a study using NAT10 deletion (*79*), a more severe perturbation that is not known to cause eIF2α phosphorylation or activation of the ISR. We hypothesize that differences in these models may arise due to the centrality of NAT10 to additional processes besides tRNA modification such as ribosome biogenesis (*6*). Evaluation of a NAT10 knockdown HeLa cell clone (*79*) revealed a more profound effect on eIF2α phosphorylation and nascent translation than caused by THUMPD1 KO (fig. S14), suggesting overlapping but distinct effects. The development of strategies for temporal modulation of NAT10 and THUMPD1 activity such as small molecule inhibition and degron-tagged models will likely be useful in differentiating first-order effects from compensatory mechanisms.

Last, our studies provide an overview of the organismal consequences of THUMPD1 deletion but do not specify in vivo mechanism or report behavioral phenotypes. Although we observe increased apoptosis in tissues showing strong phenotypes, suggesting that cell death contributes to tissue dysfunction, the precise mechanisms linking translational stress to cellular mortality in Thumpd1/Gcn2 DKO animals remain to be fully defined. Although behavioral analysis of Thumpd1 KO mice was not the focus of the current study, the molecular changes we observe in brain tissue—including increased eIF2α phosphorylation and altered gene expression—align with findings from other organismal studies of tRNA modification defects that have identified specific cognitive deficits upon extensive phenotyping (*59*). Defining these attributes will undoubtedly be helpful in applying these models to study the role of THUMPD1/tRNA acetylation in neurodevelopment.

## MATERIALS AND METHODS

### Animal models

*Thumpd1^+/−^* mice were generated by introduction of Cas9 protein and two synthetically modified guide RNAs (Synthego) into C57BL/6N fertilized eggs by microinjection. The synthetic guide RNAs were designed using sgRNA Scorer 2.0 (*80*) to target two cuts, one in exon 1 and one in exon 4. *Gcn2^+/−^* mouse was previously described (*54*) and obtained from the Jackson Laboratory (no. 008240). All mice were maintained and backcrossed on C57/BL6 background. Mice were housed at 25°C in a 12-hour light and 12-hour dark cycle. Mice were 6 to 12 weeks of age and of either sex, with age-matched littermates used as controls unless noted otherwise. Following euthanasia, tissues were isolated, flash frozen in RNAlater using liquid nitrogen, and stored at −80°C until experiments were conducted. Frederick National Laboratory for Cancer Research is accredited by AAALAC International (Association for Assessment and Accreditation of Laboratory Animal Care) and follows the NIH Public Health Service Policy for the Care and Use of Laboratory Animals. All animal experiments were approved by the NCI-Frederick Institutional Animal Care and Use Committee (approval ID: 24-437).

### Genotyping

Tail DNA from Thumpd1 mice were isolated using NaOH extraction from 10- to 12-day-old mice and subjected to PCR. PCR primer pairs used for genotyping were D9 (GCTCGTTCATGTTGCAGGTG) and E9 (CAAACTGTCGCGTACGTGTG), which amplify a short segment spanning the start of exon 1; D7 (TGAGACCACCTTCCCACAGA) and E7 (GAGTGCAAAAGACTCGCAGC), which amplify a short segment spanning the end of exon 4; and D7/E9, which amplify a product only when the segment between the guides is deleted. Genotypes were analyzed by agarose gel electrophoresis for routine genotyping, whereas MiSeq analysis was used to sequence and identify breakpoints.

### Construction of HEK-293T and HeLa *Thumpd1* KO cells

HEK-293T *Thumpd1* KO cells were generated using the CRISPR-Cas9 system. Briefly, single guide RNAs (sgRNAs) targeting protein-coding sequence of human *Thumpd1* were designed using sgRNA Scorer 2.0 (*80*). Oligonucleotides containing the 20-nucleotide spacer sequence, along with appropriate 5′ overhangs, were annealed by mixing equal quantities (∼50 pmol) of the forward and reverse oligonucleotides, heating for 2 min at 95°C, and cooling in steps of 5°C for 2 min duration until a final temperature of 25°C. Each pair of annealed oligonucleotides was ligated into the Bbs I site of the pX458 plasmid (*81*). SpCas9(BB)-2A-GFP (pX458) was a gift from F. Zhang (48138 Addgene plasmid; http://n2t.net/addgene:48138). Ligated plasmids then were transformed into the Stbl3 *Escherichia coli* strain, and colonies were grown out for large-scale plasmid preparation. Purified plasmids expressing *Thumpd1* guides 1a and 1b were cotransfected (∼1 μg) into HEK-293T (WT) cells using Lipofectamine LTX (Thermo Fisher Scientific, 15338030), and bulk GFP-positive cells were then sorted using a flow cytometer and grown for ~5 days. After assaying for protein expression by immunoblotting, candidate clones were further subjected to single-cell sorting. Confirmation of gene editing for *Thumpd1* was determined by Sanger sequencing of DNA, RNA-seq, anti-ac^4^C immuno-Northern blotting, and protein expression in whole-cell extract.

HeLa *Thumpd1* KO cell line used in this study was generated by the O’Connell lab, as previously described (*15*).

### *Thumpd1^+/−^* cross to the MMTV-PyMT metastasis model

Male FVB/N-Tg(MMTV-PyVT)634Mul/J (RRID: MGI:3032640) mice were bred to 6- to 8-week-old *Thumpd1*^*+/−*^ heterozygous female animals to generate F1 offspring. Genotyping of female animals was performed by PCR (*82*), using DNA isolated from tail biopsies taken at weaning. MMTV-PyMT positive female animals were aged until experimental end point at 120 days of age. Lungs and tumors were harvested, and tumor weight and pulmonary surface metastases were determined for each animal after euthanasia by a single investigator blinded to Thumpd1 status. Statistical significance between the two Thumpd1 genotypes was calculated using Mann-Whitney *U* tests in GraphPad Prism. All animal experiments were approved by the NCI-Bethesda Institutional Animal Care and Use Committee (approval ID: LPG-002).

### Histopathology and blood work

A full necropsy was performed to determine the spectrum of pathology in Thumpd1 KO mice. Organ weights were recorded prior to fixation for the heart, kidney, liver, brain, lung, and spleen. All tissues were fixed in 10% Neutral buffered formalin (NBF) for 72 hours and routinely processed for hematoxylin and eosin (H&E) staining and microscopic examination by a board-certified pathologist. At necropsy, blood was taken via cardiac puncture for complete blood counts (CBC), blood smear preparation, and clinical chemistry using Genesis hematology and Abaxis VetScan VS2 (Zoetis) analyzers. Full histopathology was performed on male and female WT (*n* = 6), Thumpd1 KO (*n* = 6), and Thumpd1/Gcn2 DKO (*n* = 4) mice.

### Growth conditions of human cell lines: HEK-293T and HeLa

HEK-293T cells for THUMPD1 WT, KO, or rescue were grown in Dulbecco’s modified Eagle’s medium (DMEM) (Quality Biological, 68101520) supplemented with 10% fetal bovine serum (FBS) (Avantor Seradigm, 97068-085), 2 mM l-glutamine (Thermo Fisher Scientific, 25030081), and 1% penicillin-streptomycin (Thermo Fisher Scientific, 15140122). HeLa cells for WT, THUMPD1 KO, or NAT10 KD (clone A1) (*79*) were grown under the same condition, but media were supplemented with 1 mM sodium pyruvate (Thermo Fisher Scientific, 11360-070). Cells were grown at 37°C under 5% CO_2_ and passaged at 80 to 90% confluence. Cells tested negative for mycoplasma with the LookOut MycoPlasma PCR Detection Kit following the manufacturer’s instructions (Sigma-Aldrich, MP00035).

### Extraction of total RNA from mammalian cells

HEK-293T cells were grown until ∼80 to 90% confluency before harvesting. Cells were harvested by either scraping or by the addition of trypsin-EDTA and centrifuging at 500 rcf, 2 min at room temperature. Pelleted cells were washed twice with cold phosphate-buffered saline (PBS), and pellets were stored frozen at −80°C. Total RNA from human cells was extracted using TRIzol according to the manufacturer’s protocol. One milliliter of TRIzol was used per 1 × 10^7^ cells. The RNA pellet was resuspended in water and stored at −80°C. Isolated total RNA was incubated with Turbo DNase (Invitrogen, AM2238) for 30 min at 37°C to remove any DNA contamination. Typical extractions were carried out with 1 × 10^7^ cells and yielded 400 μg of total RNA. The quality of the RNA was assessed by using the Agilent 2100 Bioanalyzer with the RNA 600 nano kit (Agilent, 5067-1511), and the concentration was determined by the Nanodrop or Qubit fluorimeter.

### Extraction of total RNA from Thumpd1 mouse tissues

The respective mouse organs were extracted from WT, heterozygous (*Thumpd1*^+/−^), and KO (*Thumpd1*^−/−^) mice and were stored in RNAlater Stabilization Solution (Thermo Fisher Scientific, AM7020) at −80°C. On the basis of the tissue type, portions of 50 to 100 mg were cut from the whole organ for RNA isolation. The tissues were minced into small pieces while soaked in RNA stabilizing agent as this improves penetration of RNA stabilizing agent and retains integrity of RNA in the tissues. The minced tissues were then transferred to a 2.0-ml prefilled (1.5 mm) zirconium homogenizer bead tube (Stellar Scientific, BS-BEBU-215) as quickly as possible while maintaining cold conditions. The bead tubes were prefilled with 1 l of chilled TRI reagent from the Direct-zol RNA Miniprep kit (Zymo Research, R2051). Samples were homogenized by bead beating with a BeadBug Microtube Homogenizer at 400 rpm. The samples in the bead tubes were given pulses four times for 30 s, incubating on ice for 30 s between each beating. To remove particulate debris from homogenized samples, the samples were left on ice for 5 min and centrifuged at 21,000 rcf for 3 min. The supernatants were transferred to nuclease-free 1.7-ml Eppendorf tubes. Total RNA was purified using the Direct-zol RNA Miniprep kit following the manufacturer’s protocol (Zymo Research, R2051), and total RNA was eluted in 50 μl of nuclease-free water. Total RNA was quantified using a Nanodrop, and the quality was checked using Agilent bioanalyzer (Agilent RNA 6000 Nano Kit, 5067-1511). The total RNA was stored at −80°C until use.

### Extraction of proteins from Thumpd1 mouse tissues

The respective mouse organs were extracted from WT, heterozygous (*Thumpd1*^+/−^), and KO (*Thumpd1*^−/−^) mice and were stored in RNAlater Stabilization Solution (Thermo Fisher Scientific, AM7020) at −80°C. Portions of 50 mg of each tissue were cut from the whole organ for protein extraction. The tissues were minced into small pieces as quickly as possible while maintaining cold conditions. The minced tissues were then transferred to a 2.0-ml prefilled (1.5 mm) zirconium homogenizer bead tube (Stellar Scientific, BS-BEBU-215). The bead tube was prefilled with 500 μl of chilled TPER tissue protein extraction reagent (Thermo Fisher Scientific, 78510) with freshly added x1 protease inhibitor cocktail (Cell Signaling Technology, 5871). Samples were homogenized by bead beating with a BeadBug Microtube Homogenizer at 400 rpm. The samples in the bead tubes were given pulses four times for 30 s, incubating on ice for 30 s between each beating. To remove particulate debris from homogenized samples, the samples were left on ice for 5 min and centrifuged at 10,000 rcf for 5 min. The supernatants were transferred to clean tubes and were sonicated on ice using 700-W QSonica Q125 sonicator with the 1/16-inch (0.15875 cm) microtip (2- × 5-s pulse, 20% amplitude, and 10-s resting on ice between pulses). Samples were centrifuged at 21,000 rcf for 30 min at 4°C. The supernatant containing the proteins was collected, leaving behind cell debris. Protein quantification was done using a Pierce BCA Protein Assay Kit (Thermo Fisher Scientific, 23225) following the manufacturer’s protocol. The proteins were stored at −80°C until use.

### Immuno-Northern blotting of ac^4^C

Total RNA was isolated from cells or mouse tissues as described above and quantified using the Qubit RNA BR assay kit (Thermo Fisher Scientific). Immuno-Northern blots were performed using Invitrogen Northern-Max reagents (Thermo Fisher Scientific). The same amount of RNA (15 μg) from each condition was aliquoted and mixed with one volume of NorthernMax-Gly Sample Loading Dye (Thermo Fisher Scientific, AM8551). These were then incubated at 65°C for 30 min and separated on a 1% agarose-1X Glyoxal Gel prepared using 10X NorthernMax-Gly Gel Prep/Running Buffer (Thermo Fisher Scientific, AM8678). Gels were run at 80 V for ~70 min or until the dye front had migrated about 3 inches (7.62 cm). Loading controls were analyzed by imaging of ethidium bromide before transfer. RNA was transferred onto Amersham Hybond-N+ membranes (Cytiva, RPN119B) using a downward capillary method as described previously (*83*). After transfer, membranes were cross-linked three times at 150 mJ/cm^2^ in a UV_254nm_ Stratalinker 2400 (Stratagene). Membranes were then blocked with 5% nonfat milk in 0.1% tris-buffered saline with Tween 20 (TBST) for 30 min at room temperature and washed three times for 5 min each in 0.1% TBST. Membranes were then incubated overnight at 4°C with the anti-ac^4^C antibody [Abcam, ab252215 (RRID: AB_2827750); 1:2000 dilution] in blocking buffer (5% nonfat milk in 0.1% TBST). Membranes were washed three times for 5 min in 0.1% TBST and then incubated with horseradish peroxidase (HRP)–conjugated secondary anti-rabbit immunoglobulin G (IgG) [Cell Signaling Technology, 7074 (RRID: 2099233); 1:10,000 dilution] in 5% nonfat milk for 1 hour at room temperature. Membranes were washed three times for 10 min each in 0.1% TBST. SuperSignal ELISA Femto Maximum Sensitivity Substrate reagent (Thermo Fisher Scientific, 37075) was added directly to the membrane, and the signal was detected via chemiluminescent imaging using an Amersham ImageQuant 800 (Cytiva, 29399482).

### Modification-induced misincorporation tRNA sequencing

#### Small RNA enrichment total RNA

Total RNA was extracted from HEK-293T cells or mouse tissues as mentioned above. Small RNA enrichment was done according to the manufacturer’s protocol using the Quick-RNA Microprep Kit (Zymo Research, R1050) using 10 to 30 μg of purified total RNA as the starting material. Small RNA was eluted in 15 to 30 μl of nuclease-free water and was quantified using a Nanodrop. The small RNA was stored at −20°C until further use.

#### Small RNA demethylation treatment using ALKBH1

For efficient and quantitative tRNA-seq, demethylation of small RNA was carried out to remove base methylations such as *N*^1^-methyladenosine (m^1^A), *N*^3^-methylcytosine (m^3^C), and *N*^1^-methylguanosine (m^1^G) present in tRNAs. The demethylation assay was done using recombinant D135S AlkB mutant enzyme expressed in-house following the method used by Zheng and co-workers (*84*). Small RNA substrate (40 pmol) was reacted with 80 pmol of ALKBH1 enzyme in a 100-μl reaction containing 300 mM KCl, 2 mM MgCl_2_, 2 mM ascorbic acid, 300 μM α-ketoglutaric acid, and 50 μM ammonium iron(II) sulfate. The mixture was incubated at room temperature for 2 hours and immediately quenched by adding EDTA to final concentration of 5 mM. RNA was purified by the addition of 300 mM sodium acetate, 1 mM EDTA, and SUPERase-In (0.1 U/μl; Invitrogen, AM2694) followed by isopropanol precipitation on dry ice for 30 min and spinning at 21,000 rcf, 4°C for 30 min.

#### Mim-tRNA-seq library preparation

Demethylated small RNA [<200 nucleotide (nt)] samples from cells or mouse tissues were used for library preparation. RNA dephosphorylation was performed with 5 U of T4 PNK (NEB, M0201L) at 37°C for 1 hour in the presence of T4 PNK buffer without adenosine triphosphate (ATP) (NEB, B0201S). For tRNA-seq libraries, a previously described 5′-phosphorylated, preadenylated adapter (oBZ407) with six randomized nucleotides at the 5′ end and a 3′ blocking group (/5Phos/AppNNNNNNCACTCGGGCACCAAGGA/3ddC) (*85*) was used. oBZ407 adapter was ligated to the RNA template using a truncated KQ T4 RNA ligase 2 (NEB, M0242L) for 1 hour at 37°C in the presence of 50% PEG-8000 (polyethylene glycol, molecular weight 8000) (NEB, B0216). Ligated RNA was gel purified and size fractionated on a Criterion Precast TBE-urea 10% denaturing polyacrylamide gel (Bio-Rad, 3450088) to enrich tRNA molecules using two markers of 40 and 80 nt and the RiboRuler low-range ssRNA (single-stranded RNA) ladder (Thermo Fisher Scientific, SM1831). Gels were stained using 1× SYBR Gold nucleic acid stain (10,000×; Invitrogen, S11494) in 1× TBE for 3 min, and 3′-adapter-ligated RNA in a size range of 60 to 100 nt was excised. Small RNA was recovered from excised polyacrylamide gel pieces by a crush and soak method followed by isopropanol precipitation with 300 mM sodium acetate, 1 mM EDTA, and SUPERase-In (0.1 U/μl; Invitrogen, AM2694) overnight at 4°C and 1.5X volume of 100% isopropanol. Next, RT was carried out using MarathonRT reverse transcriptase (Kerafast, EYU007) with RT primer oBZ408 (/5Phos/RNNNAGATCGGAAGAGCGTCGTGTAGGGAAAGAGTGTAGATCTCGGTGGTCGC/iSP18/TTCAGACGTGTGCTCTTCCGA-TCTGTCCTTGGTGCCCGAGTG). Template RNA was subsequently hydrolyzed by the addition of 1 μl of 5 M NaOH and incubation at 95°C for 3 min. Reaction products were separated from an unextended primer on Criterion Precast TBE-urea 10% denaturing polyacrylamide gel (Bio-Rad, 3450088). Gels were stained with SYBR Gold, and the region between 60 and 100 nt was excised. Circularization of purified cDNA with Circ Ligase (Biosearch Technologies, CL9021K) was carried out in 1X reaction buffer supplemented with 1 mM ATP and 50 mM MgCl_2_ for 2 hours at 60°C, followed by enzyme inactivation for 10 min at 80°C. Then, the DNA was PCR amplified using Phusion high-fidelity PCR master mix (Fisher Scientific, F531L) for 10 to 12 cycles of 10 s at 98°C, 10 s at 65°C, and 5 s at 72°C with 5′-AATGATACGGCGACCACCGAGATCTACAC-3′ and 5′-CAAGCAGAAGACGGCATACGAGAT [8-nt barcode] GTGA-CTGGAGTTCAGACGTGTGCTCTTCCG-3′ primers. PCR products were equimolarly pooled for cluster generation with additional size selection between 190 and 250 nt. The quality of the sequence libraries, size, purity, and concentration were validated using the Agilent high-sensitivity DNA 1000 kit (Agilent Technologies, 5067-4626). Sequencing was performed as single-end reads for 100 cycles on a NextSeq machine (Illumina).

### Analysis of tRNA-seq libraries and misincorporation

tRNA abundance was quantified using the mim-tRNA-seq package (https://github.com/nedialkova-lab/mim-tRNAseq). Scripts are available at https://github.com/CCBR/TRANQUIL. GitHub links are provided as additional resources and are not new to this study.

### Ribosome and disome profiling

HEK-293T WT and Thumpd1 KO cells were grown in 10-cm plates. Cells were washed with PBS once and collected with 1 ml of footprint lysis buffer [20 mM tris-HCl (pH 8.0), 150 mM KCl, 5 mM MgCl_2_, 1 mM dithiothreitol (DTT), 1% (v/v) Triton X-100, and cycloheximide (0.1 mg/ml)] with vigorous scrapping. Lysates were digested with DNase I (2 U/ml) (Thermo Fisher Scientific, AM2222) for 15 min on ice and clarified by centrifugation at 15,000 rpm for 15 min at 4°C. Lysates containing 20 μg of total RNA were digested with 750 U of RNase I (Thermo Fisher Scientific, AM2295) at 25°C for 1 hour with gentle shaking at 500 rpm and quenched by adding 200 U of SUPERase-In (Thermo Fisher Scientific, AM2694; 20 U/μl). Nuclease-treated lysates were layered on 0.9 ml of sucrose cushion [20 mM tris-HCl (pH 8), 150 mM KCl, 5 mM MgCl_2_, 1 mM DTT, and 1 M sucrose]. Ribosomes were pelleted by centrifugation in a TLA100.3 rotor at 100,000 rpm for 1 hour at 4°C, and RNA was extracted by the miRNeasy mini kit (QIAGEN). Footprints between 25 and 40 and 40 and 80 nt were size selected separately for monosome and disome library preparation, respectively. Procedures for library construction were as described previously (*85*).

For cerebellum tissue samples, lysates were prepared by homogenizing freshly harvested cerebella tissues in 3 volumes of lysis buffer [150 mM NaCl, 20 mM tris-HCl (pH 7.4), 5 mM DTT, cycloheximide (100 μg ml^−1^), 1% Triton X-100, 0.5% sodium deoxycholate, complete EDTA-free protease inhibitor, and RNasin plus (40 U mL^−1^)] using a BeadBug Microtube Homogenizer at 400 rpm. Lysates were incubated for 10 min on ice and centrifuged at 1000 rpm for 3 min at 4°C. The supernatant was removed and flash frozen in liquid nitrogen until usage. For ribosome profiling, lysates containing 40 μg of total RNA were digested with 1000 U of RNase I (Thermo Fisher Scientific, AM2295) at 25°C for 1 hour with gentle shaking at 500 rpm and quenched by adding 200 U of SUPERase-In (Thermo Fisher Scientific, AM2694; 20 U/μl). Subsequent steps were carried out as previously described, similar to the procedure for the cell lines.

#### Analysis of ribosome and disome profiling data

hg19 reference genome assembly from the University of California, Santa Cruz (UCSC) was used for human genome alignment. A human transcriptome file was generated to include canonical transcripts of known genes from the UCSC genome browser. Libraries were trimmed to remove the 3′ adapter (NNNNNNCACTCGGGCACCAAGGA), and four random nucleotides included in RT primer (RNNNAGATCGGAAGAGCGTCGTGTAGGGAAAGAGTGTAGA-TCTCGGTGGTCGC/iSP18/TTCAGACGTGTGCTCTTCCGATCTGTCCTTGGTGCCCGAGTG) were removed from the 5′ end of reads. Trimmed reads were aligned to human ribosomal and noncoding RNA sequences using STAR (*86*) with “-outFilter-Mismatch-Nover-Lmax 0.3.” Unmapped reads were mapped to the human transcriptome file with “–outFilterIntronMotifs RemoveNoncanonicalUnannotated –outFilterMultimapNmax 1 –outFilterMismatchNoverLmax 0.1” as previously described (*13*). All other analyses were performed using the software custom written in Python 3.10 and R 4.3. Scripts are available at https://github.com/NCI-RBL/Dockers/tree/main/workflows/RiboFootPrint. The GitHub link is provided as an additional resource and is not new to this study.

### Leucine and serine repeat GFP reporter assays

#### Construction of leucine and serine repeat reporter plasmids

Site-directed mutagenesis was used to insert Leu or Ser codon repeats (see table S2 for primer list and insertions) into the N terminus of a pmaxGFP (Lonza Bioscience) reporter construct. To generate each insertional GFP mutant construct, PCRs were carried out with WT pmaxGFP vector as a template, the pair of corresponding primers, and Q5 DNA polymerase (New England Biolabs, M0493S). Reactions were carried out with hot start step at 92°C for 2 min and then for 18 cycles with denaturing step at 95°C for 30 s, annealing step at 54°C for 30 s, and polymerization step at 72°C for 1 min followed by reactions incubation at 72°C for 5 min before holding at 4°C. Then, Dpn I (New England Biolabs, R0176S) was added to PCRs and incubated for 1 hour at 37°C to digest template DNA followed by enzyme deactivation at 75°C for 10 min. Dpn I–treated PCRs were used to transform chemically competent XL1-Blue cells (Agilent). The resulted colonies were selected on LB plates containing kanamycin (50 μg/ml), and plasmid DNA was prepared in LB liquid cultures containing kanamycin (50 μg/ml) using the I-Blue Mini Plasmid Kit (IBI Scientific, IB47170). Plasmids containing the correct insertions of Leu or Ser codons (as determined by sequencing using a cytomegalovirus promoter primer; table S2) were selected for GFP assays.

### GFP reporter assays

WT pmaxGFP vector and pmaxGFP vectors containing upstream Leu or Ser codon repeats were transiently transfected into HEK-293T cell line and the corresponding *THUMPD1* knockdown cell lines using jetPRIME transfection reagent (Polyplus, 101000046) as suggested by the manufacturer, in 96-well dishes in triplicate. Specifically, 100 ng of plasmid DNA samples in 10 μl of jetPRIME buffer was mixed with 0.2 μl of jetPRIME reagent, incubated 10 min at room temperature, and added to each well containing 1 × 10^4^ cells in standard DMEM (Corning). eGFP fluorescence# in live cells was measured at 48 hours posttransfection time in a SPARK microplate reader (Tecan), with excitation at 482 nm and emission at 512 nm. The GFP reporter expression was calculated as the ratio of eGFP fluorescence sample values (in triplicate) normalized to the corresponding values of the sample of the WT pmaxGFP vector transfected into HEK-293T (set to the value of 1) with the SD calculated for each sample.

### Western blotting for analysis of eIF2α phosphorylation

HEK-293T and HeLa THUMPD1 WT/KO cells and HeLa NAT10 WT/KD cells were plated at 500,000 cells per well in 6-well plates in 2 ml of complete media and allowed to adhere overnight. For ISRIB, GCN2-IN-6 treatment, and complete deprivation of glutamine, media were aspirated 24 hours after plating cells, 1500 μl of complete media with glutamine was replaced, and cells were treated dropwise with 500 μl of media containing ISRIB (Cayman Chemical, 16258) or GCN2-IN-6 (MedChemExpress, HY-130240) for final concentrations of 1.1 μM ISRIB or 50 nM GCN2-IN-6 with 0.01% dimethyl sulfoxide (DMSO). Control wells were treated with 0.01% DMSO vehicle. Cells were returned to the incubator for 30 min, and media were then aspirated. For wells indicated for glutamine starvation, cells were washed twice with media lacking glutamine. Glutamine-containing or lacking media were replaced, and cells redosed with ISRIB/DMSO as described above, and cells were returned to the incubator for 6 hours. After 6 hours, cells were washed in 1 ml of 1x cold PBS, scraped in 500 μl of PBS, transferred to a prechilled Eppendorf tube, and centrifuged (500 rcf x 5 min), and pellets were snap frozen and stored at −80°C. Cells were lysed by sonication in 1x PBS supplemented with 1x protease/phosphatase inhibitor (Cell Signaling Technology, 5872). For concentration-dependent glutamine deprivation, media were aspirated 24 hours after plating, cells were washed 1x with PBS, and media were replaced with 2 ml of media containing 2000, 200, 20, 2, 0.2, or 0 μM l-glutamine. Cells were incubated for 3 hours before harvesting and lysing cells as described above.

Total protein quantification was performed using Precision Red Protein Assay (Cytoskeleton, ADV02). SDS–polyacrylamide gel electrophoresis (PAGE) was performed using 4 to 12% Bis-Tris NuPAGE gels (Invitrogen, NP0322 and NP0323), with XCell SureLock Mini-Cells (Invitrogen, EI0002) and MES running buffer (Invitrogen, NP0002) according to the manufacturer’s protocols. Total protein (10 μg) was loaded per well, and BenchMark Pre-stained Protein Ladder was loaded on all gels (Invitrogen, 10748010). Gels were transferred by iBlot dry transfer (Invitrogen, IB1001) using nitrocellulose transfer stacks (Invitrogen, IB301001) at 20 V for 1 min, 23 V for 4 min, and 25 V for 2 min. Total protein content was visualized using Ponceau stain after washing with 5% acetic acid in water. Membranes were blocked in StartingBlock (PBS) Blocking Buffer (Thermo Fisher Scientific, 37538) for 20 min at room temperature. Membranes were then probed overnight at 4°C with antibodies for Thumpd1 [Bethyl, A304-385A-M (RRID: AB_2620838); 1:5000 or 1:2000 dilution], eIF2α [Santa Cruz Biotechnology, sc-133227 (RRID: AB_2096505); 1:1000 dilution], P-eIF2α [Abcam, ab32157 (RRID: AB_732117); 1:10,000 dilution], ATF-4 [Cell Signaling Technology, 11815S (RRID: AB_2616025); 1:1000 dilution], or vinculin [Bethyl, A302-535A-T (RRID: AB_2728768); 1:10,000 dilution], with all dilutions made in StartingBlock Blocking Buffer. Secondary antibodies were either anti-rabbit IgG HRP-linked antibody [Cell Signaling Technology, 7074S (RRID: AB_2099233)] or anti-mouse IgG HRP-linked antibody [Cell Signaling Technology, 7076S (RRID: AB_330924)] and were both incubated at 1:1000 dilutions in 5% nonfat dry milk in 1x TBST for 1 hour at room temperature. For all but one membrane, separate gels were run for eIF2α and P-eIF2α antibodies. Some membranes were reprobed with antibodies at different molecular weights. Membranes were washed at least three times with 1x TBST between antibodies and before imaging. Imaging of colorimetric and chemiluminescent signals was performed using an Amersham ImageQuant 800 (Cytiva, 29399482) and, for the chemiluminescent signal, using Lumiglo (Cell Signaling Technology, no. 7003) or SuperSignal ELISA Femto Substrate (Thermo Fisher Scientific, 37074) according to the manufacturer’s protocols.

### Procedure for cell proliferation analysis

HEK-293T and HeLa THUMPD1 WT/KO cells and HeLa NAT10 WT/KD cells were seeded at 2.5 × 10^3^ cells per well in 96-well white-walled, clear-bottomed plates. Following cell adherence, cell viability was assessed using CellTiter-Glo Cell Viability Assay (Promega, G7570). Luminescence was measured following the manufacturer’s protocol using the BioTek Cytation 5 Imaging Reader (Agilent). The time of this measurement, day 0, was recorded, and the assay was performed daily at the same time for 5 subsequent days. The assay was performed with biological replicates (*n* > 3).

### Immunohistochemistry of eIF2a and eIF2a-P

Tissue sections were stained on Leica Biosystems’ BondMax autostainer with the following conditions: heat-induced epitope retrieval with EDTA for 20 min followed by phospho-eIF2a [Cell Signaling Technology, 3398 (RRID: AB_2096481), rabbit monoclonal, 1:12] or total eIF2a [Abcam, ab169528 (RRID: AB_2819002), rabbit monoclonal, 1:400]. Positive controls included human colon carcinoma and human lung carcinoma. Isotype negative controls involved replacing primary antibody with nonclonal, isotype-matched antibody from the same species as the primary antibody. Sections of the brain, liver, and kidney were evaluated from WT (*n* = 3), Thumpd1 KO (*n* = 3), and Thumpd1/Gcn2 DKO (*n* = 3). Slides were digitalized at 20× objective (0.5 × 0.5 μm per pixel) using an Aperio AT2 scanner (Leica Biosystems) and analyzed using HALO (Indica Labs, v3.6). Appropriateness of staining and region of interest annotation were completed by a board-certified pathologist to include the cerebral cortex, hippocampus, liver, and renal cortex. The percentage of positive pixels is reported.

### In situ OPP labeling, cell culture maintenance, and proteome harvesting for gel-based fluorescence readout

HEK-293T and HeLa THUMPD1 WT/KO cells and HeLa NAT10 WT/KD cells were cultured as described above. For gel-based fluorescence detection of protein translation, three 6-cm diameter dishes (USA Scientific, CC7682-3359) were plated for each cell line with a plating density of 5.0 × 10^5^ cells. Each plate contained 5 ml of complete growth medium and returned to the cell incubator for propagation. Growth media were replaced every 2 days. Upon reaching 80% confluency, 12.5 μl of 0, 4, and 10 mM OPP stocks dissolved in DMSO (Millipore Sigma, 276855) was added in a dropwise manner to each plate and swirled in a clockwise motion to ensure even distribution of OPP. Final OPP concentrations were 0, 10, and 25 μM. To demonstrate a change in translation, a control condition was set up for each cell line that received a 15-min preincubation with 12.5 μl of 72 mM cycloheximide (EMD Millipore, 239764-10MG) stock dissolved in DMSO for a final concentration of 180 μM before the 1-hour incubation with 25 μM OPP. Plates were subsequently returned to the incubator for a 1-hour incubation. Dosing of each plate was staggered by 10-min increments to ensure that harvesting was started after the 1-hour incubation was completed. Following incubation, growth media were removed by aspiration and replaced with 1 ml of PBS. Cells were harvested by lifting cells from the plate’s surface area with a cell lifter (VWR International, 75799-938). Once cells were dislodged from the plate, the PBS solution was transferred to a 15-ml conical tube, and the cell lifting process was repeated for a total of two times. Cells were centrifuged for 5 min at 500 rcf at 4°C to form cell pellets. Following centrifugation, the supernatant was aspirated, and cells were resuspended in 1 ml of ice-cold PBS and transferred to a 1.5-ml microcentrifuge tube. Cells were then pelleted by centrifugation for 5 min at 700*g* at 4°C. The supernatant was subsequently aspirated, and cell pellets were resuspended in 100 μl of lysis buffer consisting of 1X PBS supplemented with 1X protease inhibitor cocktail (Cell Signaling Technology, 5871). Cell pellets were lysed by sonication using a 700-W QSonica Q700 sonicator (15- × 2-s pulse, amplitude 1, and 30-s resting on ice between pulses). Following sonication, lysates were centrifuged for 30 min at 4°C at 21,000 rcf. After centrifugation, supernatants were recovered, and the protein concentration was determined by Precision Red Advanced Protein Assay (Cytoskeleton, AVD02) using the manufacturer’s 96-well plate format. Lysates were subsequently diluted to a protein concentration of 1.3 mg/ml using lysis buffer and stored at −80°C.

#### Fluorogenic detection of OPP-labeled proteomes

To assess global changes in the nascent proteome, proteins (68 μl, 1.3 mg/ml) labeled by OPP were visualized by SDS-PAGE through Cu(I)-catalyzed [3 + 2] cycloaddition (CuAAC) with a fluorescent azide as previously reported (*87*). Briefly, 7 μl of a click chemistry master mixture consisting of tetramethylrhodamine (TAMRA)-azide (100 μM; 5 mM stock solution in DMSO; Millipore Sigma, 760757), TCEP (1 mM; 100 mM stock in 200 mM NaOH; Millipore Sigma, C4706-2G), tris(benzyltriazolylmethyl)amine ligand (TBTA; 100 μM; 1.7 mM stock in DMSO:*tert*-butanol 1:4; Millipore Sigma, 678937-50MG), and CuSO_4_ (1 mM; 50 mM stock in H_2_O; VWR International, BDH9312-500G) was added to each labeled proteome. Reactions were vortexed and incubated at room temperature for 1 hour in the dark. Reactions were vortexed every 20 min and returned to dark storage. Upon completion of the 1-hour incubation, the cycloaddition reaction was quenched by addition of 8.35 μl of 1000 mM DTT (Millipore Sigma, D0632). Samples were then subjected to an acetone protein precipitation to remove cycloaddition reagents. Briefly, 187 μl of acetone (Fisher Scientific, A1320) was added to each sample and incubated for 2 min at room temperature. Samples were then pelleted by centrifugation at 20,000 rcf at room temperature. Following centrifugation, the supernatant was removed and allowed to air dry for 1 min. Protein pellets were resuspended in loading buffer consisting of 4X LDS loading buffer (Invitrogen, NP0007) and 100 mM DTT. Subsequently, samples were incubated at 95°C for 10 min to dissolve the protein pellet. Fifteen microliters of each sample was analyzed by gel electrophoresis using Bis-Tris NuPAGE gels (4 to 12%, Invitrogen, NP0322) and MES running buffer (Life Technologies, NP0002) in XCell SureLock Mini-Cells (Invitrogen) according to the manufacturer’s instructions. The XCell SureLock Mini-Cell electrophoresis apparatus was connected to a PowerEase Touch 120 W Power Supply (Thermo Fisher Scientific, PS0120) at 200 V for 35 min. Gels were fixed and destained in a solution of 50/40/10% MeOH/H_2_O/AcOH overnight to remove excess probe fluorescence, rehydrated with water for 30 min, and visualized using an Amersham ImageQuant 800 imager with the IQ800 Control Software version 1.2.0.

#### Densitometry analysis of OPP-labeled proteomes

Densitometry analysis to normalize fluorescence signal to the protein loading control was done as previously reported using the ImageJ software (version 1.53t, August 2022) (*88*). Briefly, “.PNG” images of the OPP-labeled proteomes and Coomassie loading controls were uploaded to ImageJ. For a given sample, a rectangle was created to encompass a whole lane, and this process was done for all lanes analyzed. Once all lanes were selected, the lanes were analyzed using the “plot lanes” function to create a histogram of every lane. Next, the “tool” function was selected to enclose each histogram prior to selecting the “magic wand” function to integrate the area under the curve and create an “OPP fluorescence” densitometry value. This process was repeated for the Coomassie image to create a corresponding whole lane “loading control” densitometry value. The fluorescence densitometry values were normalized using the following equation: %Coomassie=(loading control)×(100). The normalized data were then plotted using the GraphPad software [version 10.2.3 (347), 21 April 2024].

### In situ OPP labeling and proteome harvesting for LC-MS analysis of protein translation

For liquid chromatography–mass spectrometry (LC-MS) detection of protein translation, four 15-cm diameter dishes were plated for each cell line with a plating density of 5.0 × 10^6^ cells. Each plate contained 15 ml of complete growth media and returned to the cell culture incubator for propagation. Growth media were replaced every 2 days. Upon reaching 80% confluency, 37.5 μl of 10 mM OPP dissolved in DMSO was added in a dropwise manner to each plate and swirled in a clockwise motion to ensure even distribution of OPP. The final OPP concentration was 25 μM, and plates were returned to cell incubator for 1-hour incubation. Dosing of each plate was staggered by 10-min increments to ensure that harvesting was started after the 1-hour incubation was complete. Following incubation, growth media were removed by aspiration and replaced with 2 ml of PBS. Cells were harvested by lifting cells from the plate’s surface area with a cell lifter. Once cells were dislodged from the plate, the PBS solution was transferred to a 15-ml conical flask, and the cell lifting process was repeated for a total of two times. Cells were centrifuged for 5 min at 500 rcf at 4°C to form cell pellets. Following centrifugation, the supernatant was aspirated, and cells were resuspended in 10 ml of ice-cold PBS and pelleted as described above. PBS was removed by aspiration, and pellets were resuspended in 1 ml of PBS prior to being transferred to a 1.7-ml microcentrifuge tube. Cells were then pelleted by centrifugation for 5 min at 700 rcf at 4°C. The supernatant was subsequently aspirated, and cell pellets were resuspended in 200 μl of lysis buffer consisting of 1X PBS supplemented with 1X protease inhibitor cocktail (Cell Signaling Technology, 5871). Cell pellets were lysed by sonication using a 700-W QSonica Q700 sonicator (15 × 2 s pulse, amplitude 35%, and 30 s resting on ice between pulses). Following sonication, lysates were centrifuged for 30 min at 4°C at 21,000 rcf. After centrifugation, supernatants were recovered, and the protein concentration was determined by Precision Red Advanced Protein Assay (Cytoskeleton AVD02) using the manufacturer’s 96-well plate format. Lysates were subsequently diluted to a protein concentration of 2 mg/ml using lysis buffer and stored at −80°C.

### RT-qPCR–based analysis of Thumpd1 mRNA

Total RNA from mouse liver tissue was isolated and quantified using a Nanodrop as described above. RT-qPCR was performed using the Luna Universal One-step RT-qPCR kit (New England Biolabs, E3005S) using a LightCycler 480 II PCR instrument (Roche). Real-time instrument was programmed with the following thermocycling protocol:

RT at 55°C for 10 min, initial denaturation at 95°C for 1 min, followed by 45 cycles of denaturation at 95°C for 10 s and extension at 60°C for 30 s.

The cycle threshold (Ct) values were obtained from the LightCycler 480 SW 1.5.1 software. Thumpd1 primers used for the RT-qPCR of the total RNA from Thumpd1 WT, KO, and heterozygous liver tissues are listed in table S2.

Melt curves were also performed to confirm the presence of a single amplicon by RT-PCR and the absence of primer dimer. The heterogeneous and Thumpd1 KO samples were normalized to the WT mouse using $2^{-∆∆C_{t}}$ relative abundance formula. The data are represented in technical triplicate (*n* = 3).

### Sanger sequencing analysis of ac^4^C in tRNA^Ser^

#### DNase treatment and demethylation

Total RNA (5 μg) was treated with 1 μl of TURBO DNase (2 U/μl) (Invitrogen AM2238) in a 50-μl reaction volume at 37°C for 30 min to remove any genomic DNA contamination. Total RNA was then purified using the RNA Clean & Concentrator-5 kit (Zymo Research, R1013) following the manufacturer’s protocol. The demethylation assay was performed as described above with modifications for total RNA. Briefly, 5 μg of total RNA (~40 pmol of tRNA) was treated with a 1:5 molar ratio of D135S mutant AlkB (200 pmol) in a reaction volume of 100 μl. The reaction buffer contained 300 mM KCl, 2 mM MgCl_2_, 50 μM (NH_4_)_2_Fe(SO_4_)_2_·6H_2_O, 300 μM 2-ketoglutarate, 2 mM l-ascorbic acid, bovine serum albumin (BSA) (50 μg/ml), and 50 mM MES buffer (pH 5.0). The reaction was incubated for 2 hours at room temperature and quenched by the addition of 5 mM EDTA. Total RNA was purified using the RNA Clean & Concentrator-5 kit (Zymo Research, R1013) following the manufacturer’s protocol.

#### NaCNBH_3_ reduction of RNA

Total RNA samples (2 to 3 μg) were next treated with the reducing agent sodium cyanoborohydride (100 mM NaCNBH_3_ in H_2_O) or water (as a control) in a final reaction volume of 100 μl. Reactions were initiated by the addition of 1 M HCl to a final concentration of 100 mM and incubated for 20 min at room temperature. Reactions were stopped by neutralizing the pH by the addition of 30 μl of 1 M tris-HCl (pH 8.0). The quenched reactions were adjusted to 200 μl with H_2_O, purified via ethanol precipitation, and washed via 70% ethanol. The pelleted RNA was dried using a SpeedVac, resuspended in H_2_O, and quantified using a Nanodrop 2000 spectrophotometer. Samples were stored at −20°C until RT was performed.

#### RT and PCR of tRNA^Ser^

An amount of ~200 to 500 ng of purified RNA from individual reactions (NaCNBH_3_ treated or water control) were mixed with 4.0 pmol of the reverse (RT) primer for tRNA^Ser^ AGA/TGA (table S2) in 1x First Strand reaction buffer (SuperScript III Reverse Transcriptase kit from Invitrogen, 18080093) to a final volume of 20 μl. The annealing reaction was done by heating to 95°C for 1 min followed by 65°C for 5 min and then transferring to ice for 1 min. After annealing, RT was performed with SuperScript III (Invitrogen, 18080093) enzyme by adding 5 mM DTT, 25 U of RNasin, 100 U of SuperScript III enzyme, and 500 μM deoxynucleotide triphosphates (dNTPs) (5 mM guanosine triphosphate, 10 mM cytidine triphosphate, 10 mM ATP, and 10 mM thymidine triphosphate). The reactions were incubated at 55°C for 60 min. Samples were quenched by increasing the temperature to 70°C for 15 min.

The cDNA products from the reactions and controls were then PCR amplified. PCRs were set up with 2 μl of the RT reaction in a 50-μl volume with 1 U of Phusion High-Fidelity DNA Polymerase (New England Biolabs M0530), 1x HF buffer, 2.5 pmol of each forward and reverse PCR primer for tRNA^Ser^ AGA/TGA (table S2), and 200 μM each dNTP. The thermocycling conditions for PCR include an initial denaturation at 95°C for 1 min, followed by 33 cycles of denaturation at 95°C for 15 s, annealing at 67°C for 30 s, and extension at 72°C for 30 s. The reaction finishes with a final extension at 72°C for 7 min before holding at 4°C.

PCR products were run on a 2% agarose gel, stained with SYBR Safe (Thermo Fisher Scientific, S33102), and visualized on an ultraviolet (UV) transilluminator at 302 nm. Bands of the desired size were excised from the gel. DNA was extracted using the NucleoSpin Gel and PCR Clean-up kit (Macherey Nagel, 740609.50) and submitted for Sanger sequencing (Genewiz) with the forward sequencing primer for tRNA^Ser^ AGA/TGA (table S2). Processed sequencing traces were viewed using the SnapGene software. The peak height for each base was measured, and the percent misincorporation was determined using the equation: Percent Misincorporation = (peak intensity of T)/(sum of C and T base peaks) × 100%. Final misincorporation values were determined by subtracting the background water control misincorporation levels from that of the corresponding reactions.

### Ac^4^C-seq

HEK-293T (American Type Culture Collection, CRL-3216) and 3T3 cells (American Type Culture Collection, CRL-1658) were cultured in 10-cm plates in DMEM with 10% FBS, penicillin (100 U/ml), and streptomycin (100 g/ml) at 37°C as described above. *S. cerevisiae* strain BY4741 was harvested in mid-log phase after growth at 30°C in standard YPD medium (1% yeast extract, 2% Bacto Peptone, and 2% dextrose). Total RNA from HEK-293T and 3T3 cells was isolated using TRIzol as described above. Total RNA was isolated from yeast using the MasterPure Complete RNA Purification Kit (VWR, MC85200) according to the manufacturer’s protocol. RNA concentrations and purity were measured by using a Nanodrop, and RNA was stored in pure water at −80°C prior to library construction.

#### Library construction

Uncharging of tRNAs was performed as previously described in QuantM-tRNA-seq (*89*). Total RNA (1 μg) for each sample was uncharged by incubating in 20 mM tris-HCl (pH 9.0) at 37°C for 45 min and subsequently neutralized by the addition of an equal volume of 20 mM sodium acetate/acetic acid (pH 4.8) with 20 mM NaCl. The uncharged total RNA samples were next size selected on a column according to the manufacturer’s protocol to maintain RNAs of <200 nt (RNA Clean and Concentrator-5 kit, Zymo Research, R1013). The size-selected RNA was next dephosphorylated and ligated with a 3′ RNA oligo containing an internal barcode, a 3-nt unique molecular index (UMI), and the Truseq R2 Illumina adapter. The barcoded samples could be pooled as described in RNAtag-seq protocol (*90*) with a 7-nt barcode and 3-nt UMI. Samples were next treated with either NaCNBH_3_ or a mock treatment of water as described in the ac^4^C-seq protocol (*23*). RT was performed using TGIRT at 42°C for 16 hours (*91*). RNA was hydrolyzed and a second ligation was performed by adding a 3′ DNA oligo (5′ to the RNA strand) containing the Truseq R1 Illumina adapter and a 6-bp UMI for BY4741/HEK-293T/3T3 or without a UMI for mouse liver samples. Barcoded primers were then used to amplify the sequencing library via PCR. Libraries were subsequently sequenced on Illumina NovaSeq 6000 platform with an SP100 kit with read lengths split evenly between R1 and R2.

#### Processing of reads, alignment, and analysis

Cutadapt (v4.2) (*92*) command (cutadapt -a AGATCGGAAGAGCACACGTCTGAAC -A AGATCGGAAGAGCGTCGTGTAGGGA -m 20) was applied to paired-end parental fastq files prior to merging of reads with BBMap (v38.90) (*93*) command (bbmerge.sh) using default settings. Merged reads were deduplicated at the sequence level based on uniqueness of the sequence present in the merged fastq files including a UMI using BBMap command (dedupe.sh) and default settings (*94*). Alignments of the merged and deduplicated reads were performed according to the bowtie2 (v2.3.5.1) parameters simplified to (bowtie2-align-s -k 100 --very-sensitive --ignore-quals --np 5 --reorder) and passed to the pipeline described in (*95*) using reference genomes sacCer3, mm10, and hg38 with annotations from the Genomic tRNA Database (*96*). Consolidated pileup tables of the aligned bam files were created by piping the output of “samtools mpileup” into cpup (https://github.com/y9c/cpup) and analysis performed to determine the cytidine to thymine misincorporation rates using custom R scripts. The GitHub link is provided as an additional resource and is not new to this study.

### RNA sequencing

Total RNA from Thumpd1 WT and KO HEK-293T cells and mouse tissues (liver, cerebellum, and testes) were extracted as described above. All the samples (*n* = 4 for mouse tissues and *n* = 3 for HEK-293T) were pooled and sequenced on NovaSeq 6000 S1 using Illumina Stranded Total RNA Prep, Ligation with Ribo-Zero Plus and paired-end sequencing. Briefly, RNA-seq FASTQ files were aligned to the reference genome using STAR (*86*) and raw counts data produced using RSEM (*97*). HEK-293T samples were aligned to GRCh38 using the GENCODE_46 GTF annotation, whereas mouse tissue samples were aligned to mm10 using the GENCODE_M21 GTF annotation. Downstream analysis and visualization were performed within the NIH Integrated Analysis Platform (NIDAP) using R programs developed by a team of NCI bioinformaticians on the Foundry platform (Palantir Technologies). The RSEM count matrices were filtered for low counts (<1 cpm) and normalized by quantile normalization using the limma package (*98*). Differentially expressed genes were calculated using limma-Voom (*99*). GSEA was performed using fgsea package (*100*)^.^

### Proteomic analysis

#### Lysis, digestion, and TMTpro labeling

Each cell pellet was lysed in 500 μl of EasyPep Lysis buffer (Thermo Fisher Scientific, PN A45735) and treated with 2 μl of universal nuclease (Thermo Fisher Scientific, PN 88700). The protein concentration was determined by the BCA method, and 100 μg was taken from each condition for digestion. Samples were adjusted to 100 μl of total with lysis buffer and treated with 50 μl each of reducing solution and alkylating solution provided with the EasyPep kit (Thermo Fisher Scientific, A40006). Incubated for 1 hour in the dark at 25°C and then made four aliquots of 40 μl (20 μg) for each condition and added 60 μl of trypsin/LysC (13 ng/μl; provided with the EasyPep kit) and incubated at 37°C overnight for a total of 19 hours at which point 20 μl of TMTpro 18-plex label (5 μg/μl; Thermo Fisher Scientific, PN A52045) was added to samples and incubated for 1 hour at 25°C. Excess TMTpro was quenched with 20 μl of 5% hydroxylamine and 20% formic acid (FA) for 10 min, and samples were then combined. Samples were cleaned using EasyPep mini columns provided with the EasyPep kit as described in the manual. Eluted peptides were dried in SpeedVac.

#### Offline fractionation and LC-MS analysis of peptides

TMTpro-labeled peptides were fractionated by high-pH reversed-phase LC using a Waters Acquity UPLC system with a fluorescence detector (Waters, Milford, MA) using a 150 mm–by–3.0 mm Xbridge Peptide BEMTM 2.5-μm C18 column (Waters, MA) operating at 0.35 ml/min. The dried peptides were reconstituted in 50 μl of mobile phase A (10 mM ammonium formate, pH 9.3) and eluted from the column in mobile phase B (10 mM ammonium formate and 90% ACN, Thermo Fisher Scientific). The peptides were eluted using gradient elution of 10 to 50% phase B (1.5 to 60 min) followed by 50 to 90% phase B (60 to 65 min). Sixty-five fractions were collected, and the fractions were then consolidated into 12 pools based on the chromatogram intensity and vacuum centrifuged to dryness. Each fraction was resuspended in 50 μl of 0.1% FA, and 10 μl was analyzed using a Dionex U3000 RSLC in front of a Orbitrap Eclipse (Thermo Fisher Scientific) equipped with an EasySpray ion source. Solvent A consisted of 0.1% FA in water, and Solvent B consisted of 0.1% FA in 80% ACN. Loading pump consisted of Solvent A and was operated at 7 μl/min for the first 6 min of the run and then dropped to 2 μl/min when the valve was switched to bring the trap column (Acclaim PepMap 100 C18 HPLC Column, 3 μm, 75-μm inside diameter, 2 cm, PN 164535) in line with the analytical column EasySpray C18 HPLC Column (2 μm, 75-μm inside diameter, 25 cm, PN ES902). The gradient pump was operated at a flow rate of 300 nl/min. Each run used a linear LC gradient of 5 to 7% B for 1 min, 7 to 30% B for 83 min, 30 to 50% B for 25 min, and 50 to 95% B for 4 min, holding at 95% B for 7 min, and then reequilibration of the analytical column at 5% B for 17 min. MS acquisition used the TopSpeed method with a 3-s cycle time and the following parameters: Spray voltage was 1800 V and ion transfer temperature was 275°C. MS1 scans were acquired in the Orbitrap with a resolution of 120,000, automatic gain control (AGC) of 4 × 10^5^ ions, and max injection time of 50 ms, mass range of 400 to 1600 *m/z* (mass/charge ratio); MS2 scans were acquired in the Orbitrap using method with a resolution of 50,000, AGC of 1.25 × 10^5^, max injection time of 86 ms, Higher Energy Collision Dissociation (HCD) energy of 38%, isolation width of 0.4 Da, intensity threshold of 2.5 × 10^4^, and charges 2 to 5 for MS2 selection. Advanced peak determination, monoisotopic precursor selection, and EASY-IC for internal calibration were enabled, and dynamic exclusion was set to a count of 1 for 15 s.

#### Database search and postprocessing analysis

MS files were searched together with Proteome Discoverer 2.4 using the Sequest node. Data were searched against the UniProt Human database from February 2020 using a full tryptic digest, two max missed cleavages, minimum peptide length of 6 amino acids, and maximum peptide length of 40 amino acids, an MS1 mass tolerance of 10 parts per million, MS2 mass tolerance of 0.02 Da, and fixed modifications for TMTpro (+304.207) on lysine and peptide N terminus and carbamidomethyl (+57.021) on cysteine and variable oxidation on methionine (+15.995 Da). Percolator was used for false discover rate (FDR) analysis, and TMTpro reporter ions were quantified using the Reporter Ion Quantifier node and normalized using the total peptide intensities of each channel. The log_2_FC (median of groups) and *P* values [analysis of variance (ANOVA)] were calculated within the PD2.4 software. The FDR was set to <1%. Proteins with a *P* value of <0.05 and log_2_FC cutoffs of >0.6 and <−0.6 were considered differentially expressed. The TMTpro channel assignment for conditions was as follows: WT replicates: 126, 127N, 127C, and 128N; KO replicates: 128C, 129N, 129C, and 130N; and rescue replicates: 130C, 134N, 134C, and 135N.

### Northern blotting–based analysis of tRNA charging

#### Total RNA isolation under acidic conditions

Total RNA for Northern immunoblots was prepared under acidic conditions to preserve aminoacylation essentially as described by Varshney (*101*) and applications of this approach by Chernyakov and co-workers (*102*). WT or *THUMPD1* knockdown HEK-293T cells grown to about 70 to 75% confluency on 100-mm dishes were quickly washed with cold 0.3 M sodium acetate and 10 mM EDTA (pH 4.5). Then, 0.25 ml of wash buffer was added to the cells followed by the addition of 0.75 ml of TRIzol reagent (Life Technologies) saturated with the wash buffer and thorough pipetting of cell suspension to promote cell lysis. After 5 min, the cell lysate was transferred to a microfuge tube and 0.2 ml of chloroform was added, and the suspension was vortexed and incubated for 2 min to allow for phase separation and centrifuged at 12,000 rcf for 15 min at 4°C. The aqueous phase was transferred to a new tube and RNA was precipitated by the addition of two volumes of cold 100% ethanol, incubating on dry ice for 30 min, and centrifugation at max speed for 15 min at 4°C. RNA pellets were washed with cold 70% ethanol, recentrifuged, air dried for 10 min, and resuspended in 10 mM sodium acetate and 1 mM EDTA (pH 4.5). RNA concentrations were measured using a NanoDrop Microvolume Spectrophotometer (Thermo Fisher Scientific). All RNA samples were prepared in triplicate.

#### Northern immunoblot analysis of tRNA aminoacylation

RNA samples (10 μg of total RNA) prepared under acidic conditions were resolved on 1-mm-thick 6.5% polyacrylamide gel (acrylamide–to–bis-acrylamide ratio of 19:1) containing 8 M urea and 0.1 M sodium acetate (pH 4.5). Each sample was mixed with an equal volume of acidic RNA loading dye [0.1 M sodium acetate (pH 4.5), 8 M urea, 0.05% bromophenol blue, and 0.005% xylene cyanol] and ran at 450 V for about 20 hours in a cold room. The samples were loaded in triplicate. Deacylated RNA controls were prepared by incubating the samples in 0.1 M tris-HCl (pH 9.0) and 1 mM EDTA for 30 min at 37°C followed by ethanol precipitation and dissolving the RNA pellets in 10 mM sodium acetate and 1 mM EDTA (pH 4.5). After separation on the gel, the portion of the gel containing tRNA was cut and RNA was transferred to a Hybond-N^+^ nylon membrane (Cytiva) using Bio-Rad Protean II electrophoresis cell in 1× tris-acetate-EDTA (TAE) buffer at 15 V for 1 hour in a cold room. The membrane was rinsed in fresh 1× TAE buffer and air dried for about 30 min, and RNA was UV cross-linked at 120,000 mJ/cm^2^ in Stratalinker UV cross-linker (Stratagene). After RNA cross-linking, the Nylon membrane was soaked in 2× SSC for 1 min then prehybridized in ULTRAhyb Ultrasensitive Hybridization Buffer (Ambion) for 1 hour at 42°C using a Hybaid H-9360 hybridization oven followed by hybridization with 50 to 100 pmol of biotinylated oligonucleotide probe specific to one of the tRNAs of interest (table S2) for 12 hours at 42°C. The membrane then was washed twice with 2× SSC and 0.1% SDS and once with 2× SSC for 30 min at 42°C each time. After that, the membrane was soaked for 1 min in 1× PBS containing 0.05% Tween 20 followed by blocking in 5% BSA dissolved in the same buffer for 30 min at room temperature. Then, the membrane was incubated with HRP-conjugated streptavidin (BioLegend, 1:1000 dilution in 1× PBS and 0.05% Tween 20, 405210) for 2 hours at room temperature and washed twice with 1× PBS and 0.05% Tween 20 and once with 1× PBS 20 min each time. The membrane was treated for 5 min with Thermo Fisher Scientific SuperSignal West Pico PLUS Chemiluminescent Substrate (0.1 ml/cm^2^), and tRNA bands on membrane were visualized using a Bio-Rad ChemiDoc XRS+ imager and Image Lab 4.1 software.

### qPCR-based tRNA charging assay

The tRNA charging assay was performed as previously described by Pavlova and co-workers (*103*). Briefly, HEK-293T THUMPD1 WT and KO cells were grown in DMEM as described above and washed with cold PBS and lysed by adding 1 ml of TRIzol (Thermo Fisher Scientific, 15596026) to the plate and incubating for 5 min on ice. Lysates were then collected and mixed with 200 μl of chloroform in an Eppendorf tube by shaking vigorously for 20 s. After centrifuging for 15 min at 18,600 rcf at 4°C, the top fraction was collected, and the total RNA was precipitated with 2.7x volumes of cold ethanol in the presence of 2 μl of GlycoBlue Coprecipitant (Thermo Fisher Scientific, AM9515) overnight at 4°C. The pellet was resuspended in 300 μl of tRNA precipitation buffer [0.3 M acetate buffer (pH 4.5) and 10 mM EDTA] and pelleted again by adding 2.7x volumes of cold ethanol, incubating at −20°C overnight, and centrifuging for 30 min at 18,600 rcf at 4°C. The pellet was washed with 80% ethanol and resuspended in 32 μl of tRNA resuspension buffer [10 mM acetate buffer (pH 4.5) and 1 mM EDTA] before determining the concentration via a Nanodrop. For the oxidation treatment, 2 μg of RNA from each sample was used. Oxidization reactions were done by treating the RNA with 0.2 M NaIO_4_ in sodium acetate buffer (pH 4.5) for 20 min at room temperature in the dark. For the controls, 0.2 M NaCl was used. Reactions were quenched by adding 2.2 μl of 2.5 M glucose and incubating for 15 min at room temperature in the dark. Into each glucose-quenched reaction, 1 μl of yeast tRNA^Phe^ (Sigma-Aldrich, R4018) solution (7 ng/μl stock in water) was added to serve as a spike-in control and the RNA was precipitated with ethanol as previously described. Next, to facilitate the deacylation, pelleted RNA was resuspended in 100 μl of 50 mM Tris (pH 9) and incubated at 37°C for 45 min. The reactions were quenched with 100 μl of tRNA quench buffer [50 mM Na acetate buffer (pH 4.5) and 100 mM NaCl], and RNA was pelleted by ethanol precipitation. The pellet was resuspended in 10 μl of water, and the concentration was measured using a Nanodrop. Concentrations in all the tubes were adjusted to the same level by adding water. Next, the adapter ligation step was carried out with a 5′-adenylated DNA adaptor (5′-/5rApp/TGGAATTCTCGGGTGCCAAGG/3ddC/-3′) and ~380 ng of RNA, using T4 RNA ligase 2, truncated KQ (NEB, M0373) at 18°C, overnight. Then, the CSQ_RT primer (5′-GCTGCCTTGGCACCCGA-GAATTCCA-3′) was annealed (30 s at 90°C, 5 min at 65°C, and immediately put on ice for 1 min) to the adapter region of tRNA before carrying out the RT reaction with SuperScript RT IV (Thermo Fisher Scientific, 18090050) according to the manufacturer’s protocol. The synthesized cDNA was diluted (1:10), and 2.5 μl of each reaction was used to set up each qPCR with tRNA isodecoder-specific primers using 2x SYBR Green mix (Life Technologies, 4368702). The forward primer matched the 5′ end of the tRNA, and the reverse primer covered the junction between the 3′ end of the tRNA and the ligated adaptor. The primer pairs used are listed in table S2.

LightCycler 480 II PCR (Roche) instrument was used for the qPCR step with the following thermocycling protocol:

Initial denaturation at 95°C for 1 min, followed by 45 cycles of denaturation at 95°C for 10 s and extension at 60°C for 30 s.

The Ct values were obtained from the LightCycler 480 SW 1.5.1 software. The average Ct value for each reaction and control was calculated from two technical qPCR replicates for each of the three biological replicates. Last, the charged tRNA fraction was calculated by normalizing the average ^tRNA^Ct to the yeast-Phe tRNA using the following formula

$$\begin{array}{c} ∆∆\text{Ct}=\left(∆\text{Ct}\;\text{Leu}-∆\text{Ct}\;\text{yphe}\right)\text{rxn}-\\ \left(∆\text{Ct}\;\text{Leu}-∆\text{Ct}\;\text{yphe}\right)\text{con}\;\text{charged fraction}=2^{-\left(∆∆\text{Ct}\right)} \end{array}$$

### Analysis of mTOR activation in THUMPD1 KO cells

HEK-293T THUMPD1 WT/KO cells were plated (3 × 10^6^ cells) into 100-mm tissue culture plates with 10 ml of complete medium (10% FBS, DMEM, and 1% penicillin-streptomycin) and allowed to adhere for 48 hours. Next, cells were starved with DMEM lacking amino acids (Thermo Fisher Scientific, ME120086L1) for 1 hour and/or refed with complete medium for 1 hour. For inhibitor treatments, the medium was aspirated out and 10 ml of DMEM complete medium with DMSO, DMSO + rapamycin (10 nM), or DMSO + AZD2014 (1 μM) was replaced for 1 hour. After incubation cells were collected with a cell scraper and lysed in cold NP-40 lysis buffer (50 mM Tris, 150 mM NaCl, and 1.0% NP-40 at pH 8.0). The protein lysate (25 μg) was separated in Novex WedgeWell 10 to 20% tris-glycine gels (Thermo Fisher Scientific, XP10202BOX) for proteins smaller than 100 kDa or 3 to 8% tris-acetate gels (Thermo Fisher Scientific, EA03785BOX) for proteins larger than 100 kDa. Proteins were transferred to polyvinylidene difluoride membranes (Bio-Rad, 1620177), incubated with primary antibodies overnight at 4°C, and then incubated with secondary antibodies at room temperature for 1 hour in 1x TBST + 5% blocking (Bio-Rad, 1706404). Membranes were incubated with SuperSignal West Pico Plus Chemiluminescent Substrate (Thermo Fisher Scientific, 34578). All antibodies from Bethyl Laboratories were as follows: Raptor [A300-553A (RRID: AB_2130793)] and Rictor [A300-459A (RRID: AB_2179967)]. All antibodies from CST were as follows: (Thr^389^) p-p70 S6 kinase [9234S (RRID: AB_2269803)], S6K1 [2708S (RRID: AB_390722)] (Ser^473^) p-AKT [4058S (RRID: AB_331168)], total AKT [4685S (RRID: AB_10698888)], (Ser^235/236^) p-S6 ribosomal protein [4858S (RRID: AB_916156)], (Ser^240/244^) p-S6 ribosomal protein [2215S (RRID: AB_331682)], S6 ribosomal protein [2217S (RRID: AB_331355)], (Thr^37/46^) p–4E-BP1 [9459S (RRID: AB_330985)], (Ser^65^) p–4E-BP1 [9451S (RRID: AB_330947)], 4E-BP1 [9452S (RRID: AB_331692)], and GAPDH [2118S (RRID: AB_561053)]. Antibodies p-S6, S6, and 4E-BP1 were used at 1:3000 dilution and the remainder at 1:1000 dilution in 5% BSA in 1× TBST buffer with 0.04% sodium azide. Secondary antibodies for immunoblot analysis: 1:10,000 dilution for an α-mouse IgG HRP conjugate [Promega, W4021 (RRID: AB_430834)] and 1:20,000 dilution for an α-rabbit IgG HRP conjugate [Promega, W4011 (RRID: AB_430833)].

### Northern blotting analysis of tRNA^Leu/Ser^ in THUMPD1 WT/KO HEK-293T cells

Total RNA was extracted using TRIzol reagent (Ambion) according to the manufacturer’s instructions. For each sample, 1.5 μg of total RNA, along with a ^32^P-labeled Decade marker (Ambion), was resolved on 20% (w/v) acrylamide/8 M urea gels via electrophoresis. The RNA was subsequently transferred onto a Hybond-N membrane (Amersham Pharmacia Biotech) using a semidry transfer system at 5 W for 30 min. Following the transfer, the membrane was UV cross-linked to ensure RNA immobilization and prehybridized with PerfectHyb Plus Hybridization Buffer (Sigma-Aldrich) at 37°C for 10 min. The membrane was then hybridized overnight at 37°C with ^32^P-labeled probes (see table S2 for probe sequences). After hybridization, the membrane was washed three times for 15 min each with 2× SSC containing 0.1% SDS at 37°C. For detection, the membrane was exposed to an Imaging Screen-K (Bio-Rad) for 1 hour, and the signal was captured using the Typhoon Trio Imaging System (GE Healthcare). Quantification of the Northern blot results was performed using the Quantity One software (Bio-Rad).

### Quantification of apoptosis using CC3 immunohistochemistry and TUNEL

Formalin-fixed testes, brain, and liver tissues were submitted for H&E, cleaved caspase-3 immunohistochemistry (CST 9661, 1:800, HIER Citrate), and TUNEL assay. Positive controls included mouse testes and thymus tissues. IHC staining was performed on Leica Biosystems’ BondMax autostainer with the following conditions using the Epitope Retrieval 1 (Citrate) and Bond Polymer Refine Detection Kit (Leica Biosystems, DS9800) with omission of the PostPrimary Reagent. Isotype control reagents were used in place of the primary caspase-3 antibody for negative controls. Slides were digitalized at 20× objective (0.5 × 0.5 μm per pixel) using an Aperio AT2 scanner (Leica Biosystems) and analyzed using HALO (Indica Labs, v3.6). Appropriateness of staining and region of interest annotation were completed by a board-certified pathologist to include target tissues and exclude necrosis, nontarget tissues, and histology artifacts. Quantification of positivity was performed using digital image analysis, which included identifying stain vectors, optimizing cell detection algorithms, and thresholding chromogenic IHC staining based on positive and negative controls. The percentage of positive cells is reported for each stain.

## Supplementary Materials

### The PDF file includes:

Figs. S1 to S14 Full uncropped gels and Western blots Legends for tables S1 and S2 Legends for data S1 to S12
