# Supplementary material for: Transfer RNA acetylation regulates in vivo mammalian stress signaling
Source: Sci Adv. 2025 Mar 19;11(12):eads2923. doi: 10.1126/sciadv.ads2923 (PMC11922055; doi:10.1126/sciadv.ads2923)
Supplement: Supplementary file 1 — Figs. S1 to S14 Full uncropped gels and Western blots Legends for tables S1 and S2 Legends for data S1 to S12 [file sciadv.ads2923_sm.pdf]

Science Advances
AAAS

Supplementary Materials for

Transfer RNA acetylation regulates in vivo mammalian stress signaling

Supuni Thalalla Gamage *et al.*

Corresponding author: Jordan L. Meier, jordan.meier@nih.gov; Colin Chih-Chien Wu, colin.wu2@nih.gov; Mitchell R. O'Connell, mitchell_oconnell@urmc.rochester.edu

Sci. Adv. **11**, eads2923 (2025)
DOI: 10.1126/sciadv.ads2923

The PDF file includes:

Figs. S1 to S14
Full uncropped gels and Western blots
Legends for tables S1 and S2
Legends for data S1 to S12

Other Supplementary Material for this manuscript includes the following:

Tables S1 and S2
Data S1 to S12

SUPPLEMENTARY FIGURES

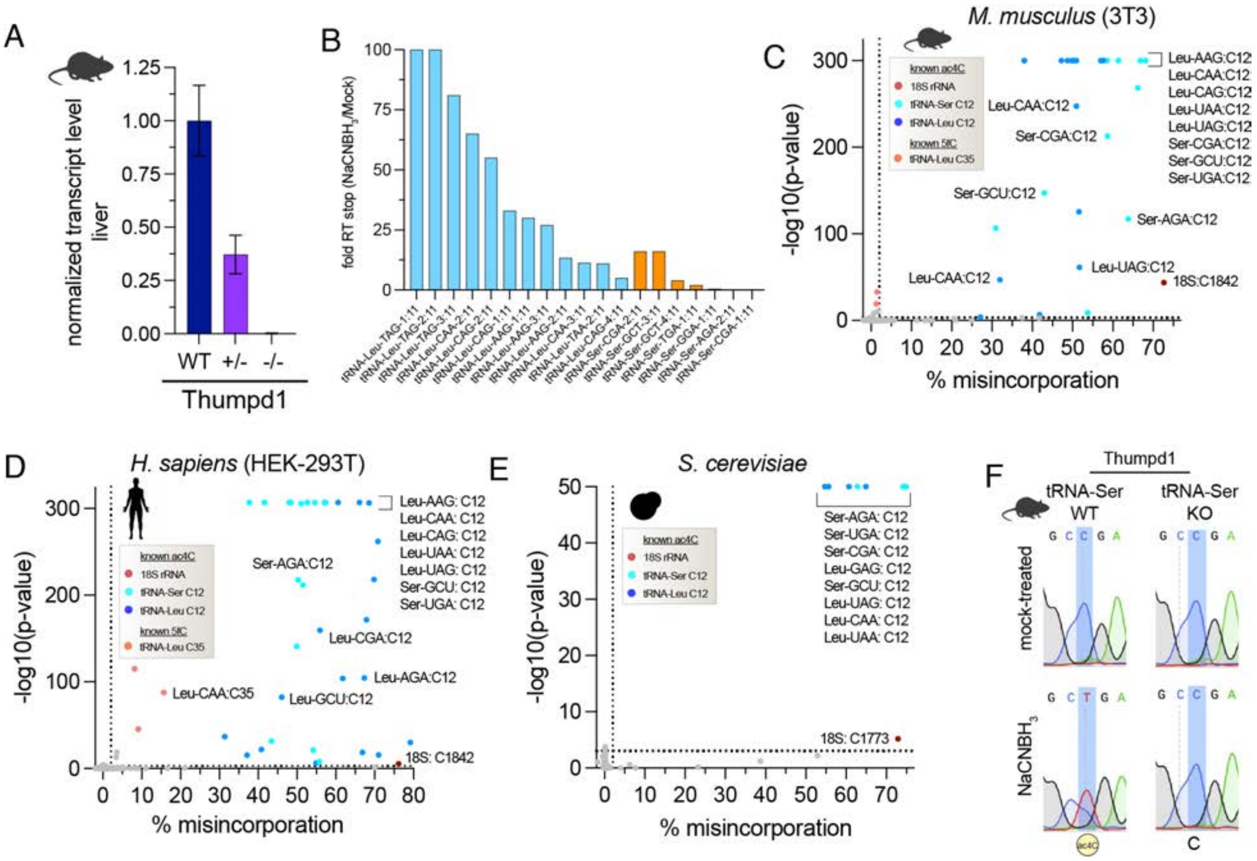

Figure S1. THUMPD1 knockout validation and ac^{4}C mapping reveals tRNA modification patterns across species. (a) Quantitative real-time PCR-based analysis of *Thumpd1* gene expression in RNA isolated from liver of *Thumpd1*^{WT}, *Thumpd1*$^{+/-}$, or *Thumpd1*$^{-/-}$ lines. Data represent the average of n=3 biological replicates. (b) Analysis of RT stops in tRNA^{Leu} (blue) and tRNA^{Ser} (orange). Fold RT stop was estimated by comparing the number of reads starting at C11 in $NaCNBH_3$-treated ('starts.sample') versus control ('starts.control'). At positions where no stops were observed in the control the ratio was arbitrarily set to 100. (c) Distribution of ac^{4}C in murine (3T3) small RNA fraction. (d) Distribution of ac^{4}C in *H. sapiens* (HEK-293T) small RNA fraction. (e) Distribution of ac^{4}C in *S. cerevisiae* small RNA fraction. Values for c-e were calculated from the 'C2T.MRD' and 'pval.CT2' columns which correspond to the C -> T misincorporation rate and C->T p-value, respectively in Data S2. Nucleotide with a 'pval.CT2' of 0 were graphed on the y-axis at the value corresponding to the lowest calculatable p-value. (f) Sanger sequencing based ac^{4}C sequencing confirms loss of ac^{4}C in murine tRNA upon Thumpd1 knockout.

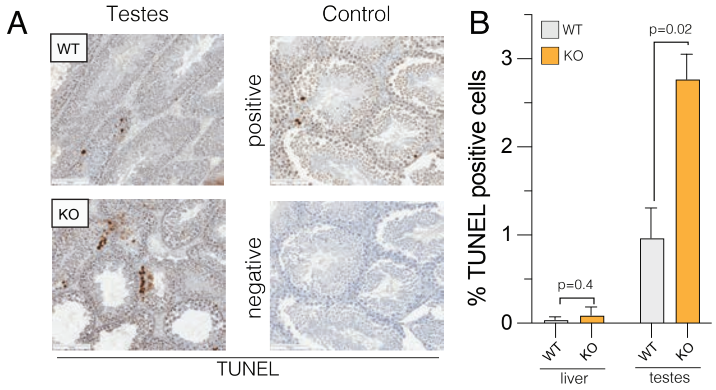

Figure S2. THUMPD1 knockout leads to increased apoptosis in mouse tissues. TUNEL analysis of tissues isolated from Thumpd1 WT/KO mice. (a) TUNEL staining of fragmented DNA in apoptotic cells within testes tissue isolated from age-matched WT and Thumpd1 KO mice. (b) Quantification of percent positive cells in mouse liver and testes tissues. Significance was analyzed by two-tailed Student's *t* test. Data represent the average of n=3 biological replicates.

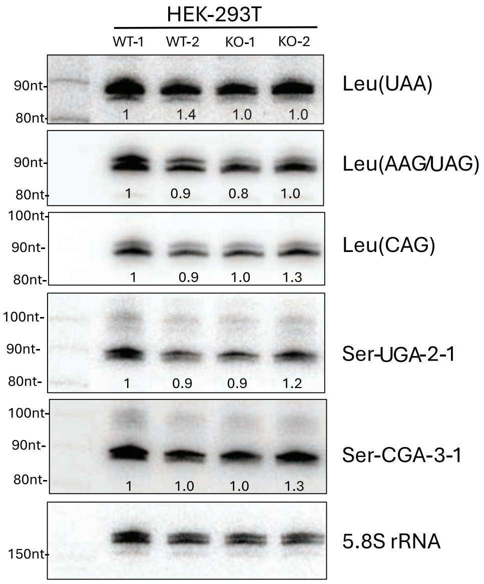

Figure S3. Northern blotting examines tRNA levels upon THUMPD1 deletion. Northern blotting analysis of tRNA$^{Leu/Ser}$ in THUMPD1 WT/KO HEK-293T cells. The identity of individual tRNA species detected by each probe are indicated. Quantifications reflect the intensity of each tRNA signal (e.g. Leu(UAA)) relative to 5.8S rRNA (loading control), with the leftmost lane (WT-1) set equal to 1. Biological replicates are loaded in adjacent lanes.

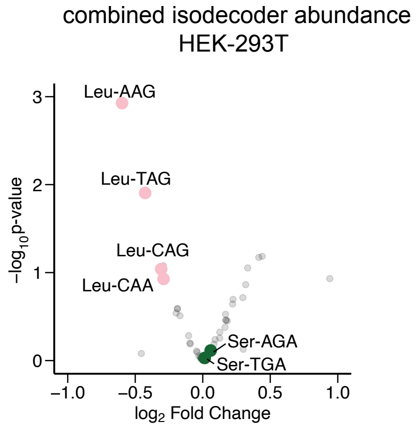

Figure S4. Loss of THUMPD1 affects anticodon-specific tRNA abundance. mim-tRNA-Seq analysis of tRNA levels at the anticodon level from WT and THUMPD1 KO HEK-293T cells. Each dot represents the aggregated tRNA levels from all isodecoders that decode each codon.

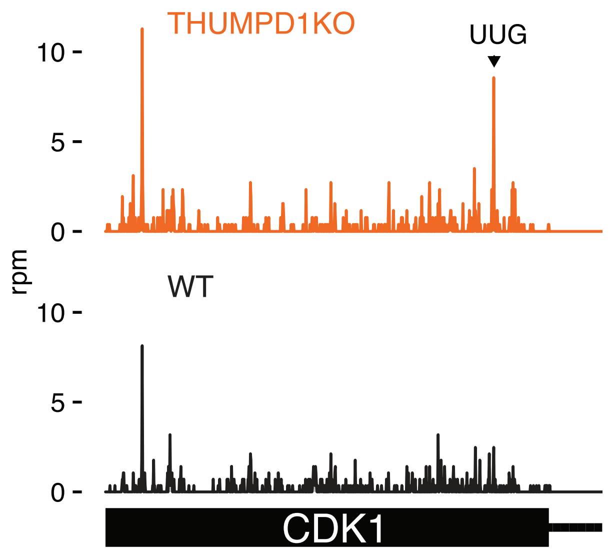

Figure S5. Ribosome stalling at leucine codons maintains downstream translation. Ribosome footprints in CDK1 gene indicate that stalling at Leu codons in KO cells does not limit downstream ribosome occupancy.

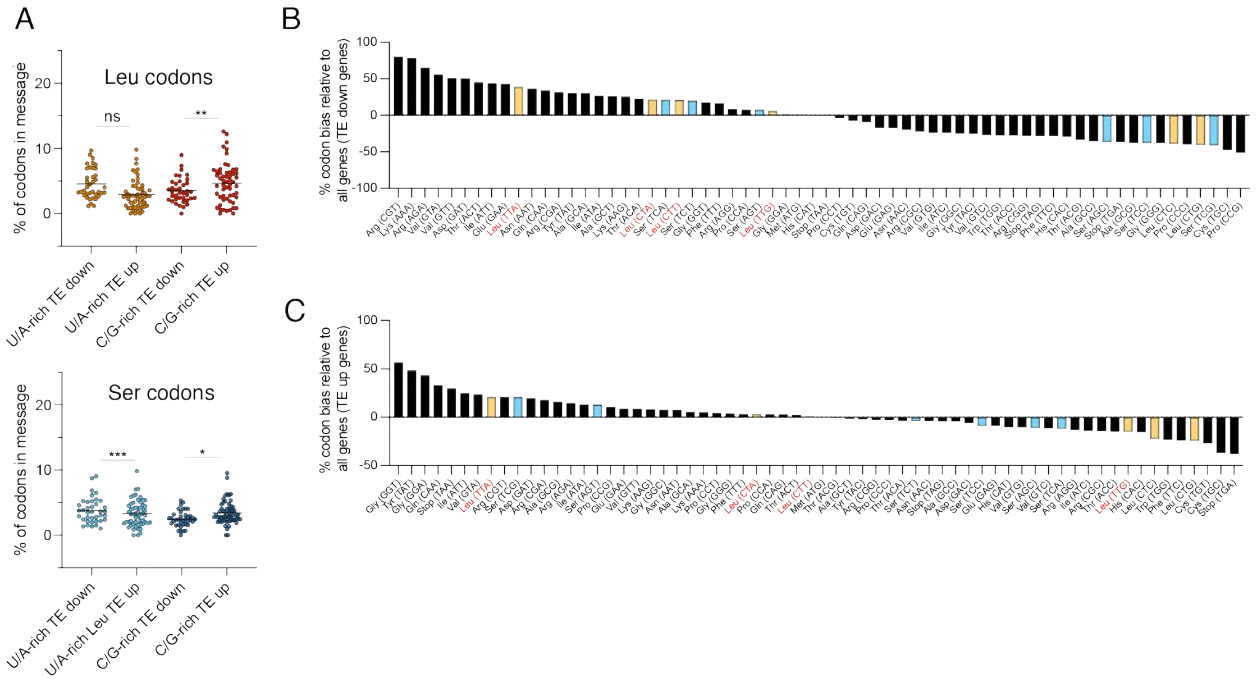

Figure S6. THUMPD1 loss differentially affects translation of transcripts based on codon composition. (a) Top: Comparative analysis of U/A-rich Leu codons (UUA, CUA, CUU, UUG) and C/G-rich Leu codons (CUG, CUC) in genes with increased (TE up) or decreased (TE down) translational efficiency in THUMPD1 KO HEK-293T cells. Bottom: Comparative analysis of U/A-rich Ser codons (UCA, UCU, AGU) and C/G-rich Ser codons (AGC, UCC, UCG) in genes with increased (TE up) or decreased (TE down) translational efficiency in THUMPD1 KO HEK-293T cells. Each point represents the combined codon content of a single TE up or TE down transcript. Significance was analyzed by two-tailed Student's *t* test (ns = not significant, * = $P < 0.05$, ** = $P < 0.01$, and *** = $P < 0.001$). (b) Analysis of codon bias in TE down transcripts relative to all CCDS-defined consensus coding sequences. A positive value indicates an increased proportion of the codon is present in TE down sequences. Leu codons = orange, Ser codons = blue, U/A-rich Leu codons are labeled on the x-axis in red. (c) Analysis of codon bias in TE up transcripts relative to all CCDS-defined consensus coding sequences. A positive value indicates an increased proportion of the codon is present in TE up sequences. Leu codons = orange, Ser codons = blue, U/A-rich Leu codons are labeled on the x-axis in red.

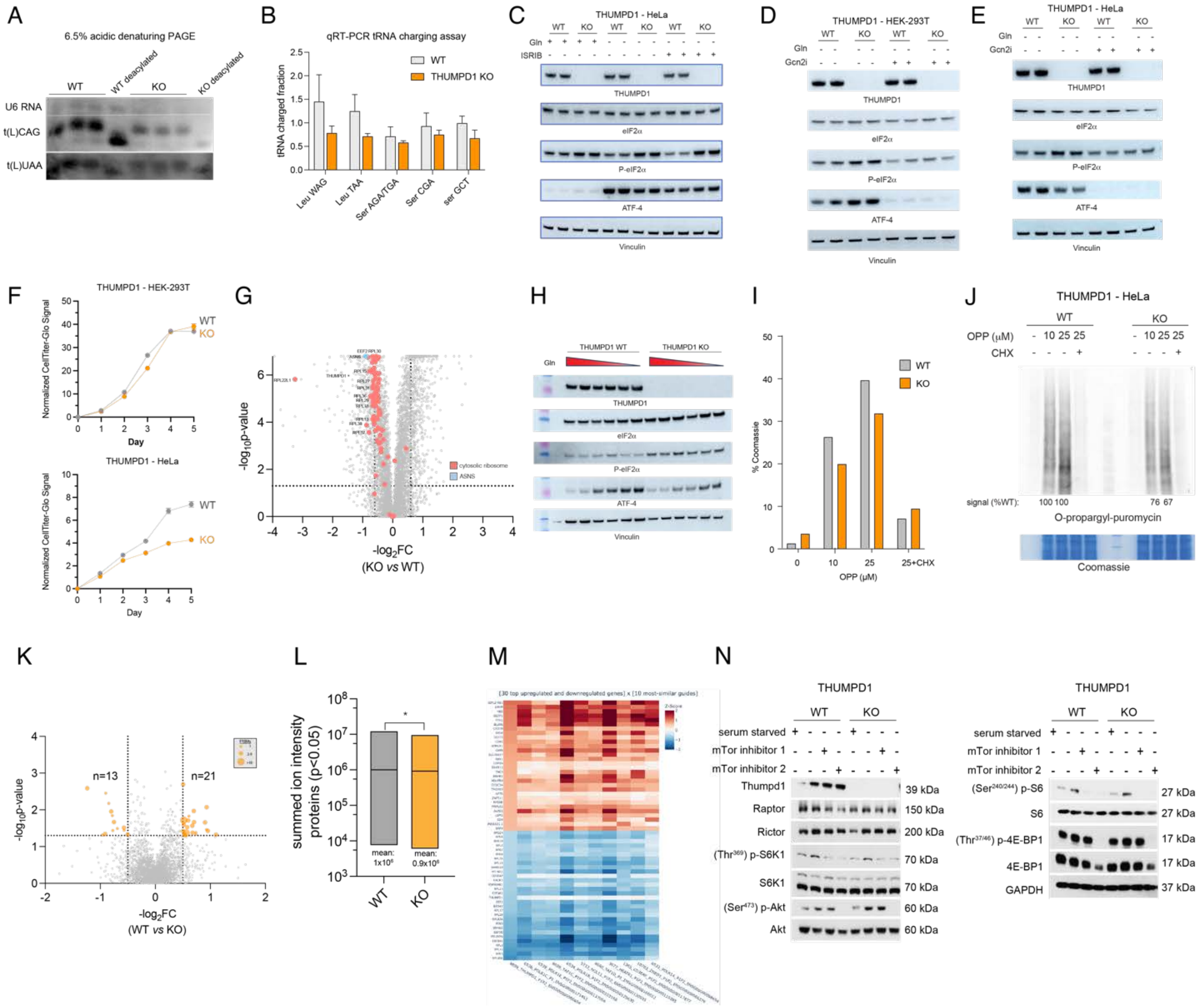

Figure S7. THUMPD1 deletion impacts translation through GCN2-dependent mechanisms. (a) Acidic denaturing PAGE analysis of tRNA charging in WT or THUMPD1 KO HEK-293T cells. Data are representative of n=2 biological replicates. (b) RT-qPCR analysis of tRNA charging in WT or THUMPD1 KO HEK-293T cells. Values for WT and KO tRNA pairs were not significant as analyzed by two-tailed Student's *t* test ($P > 0.05$). Data are representative of n=2 biological replicates. (c) Analysis of eIF2α phosphorylation in HeLa WT and THUMPD1 KO cells. Amino acid deprivation (-Gln) and ISRIB (1 µM) were used to induce translational stress. Biological replicates are loaded in adjacent lanes. (d, e) Analysis of eIF2α phosphorylation in HEK-293T (d) and HeLa (e) THUMPD1 WT/KO cells following Gcn2 (50 nM GCN2-IN-6) inhibition. Amino acid deprivation (-Gln) was used to induce translational stress. Biological replicates are loaded in adjacent lanes. (f) Effect of THUMPD1 KO on cell growth in HEK-293T and HeLa. Data are representative of n>3 biological replicates. (g) Ribosomal proteins (red) are downregulated in THUMPD1 KO HEK-293T cells. The ATF-4 target ASNS (blue) is also downregulated, suggesting the ISR is not activated by THUMPD1 KO. Values are derived from n=3 biological replicates. (h) THUMPD1 KO does not alter the threshold for glutamine-dependent activation of ATF-4. Glutamine concentrations (left to right): 2 mM, 0.2 mM, 0.02 mM, 0.002 mM, and no glutamine. (i) Gel densitometry analysis of fluorescence signal from treatment of THUMPD1 WT and KO HEK-293T with *O*-propargyl puromycin (OPP) followed by click chemistry to a fluorescent azide. The percent of the fluorescent signal relative to the Coomassie signal was calculated and used to produce the relative values given in Fig. 4g.

Data are representative of n=2 biological replicates. (j) Analysis of global translation in HeLa THUMPD1 WT and KO cells. O-propargyl puromycin (OPP) was used to label nascent transcripts, which were then ligated to a fluorophore-azide and visualized via SDS-PAGE. (k) LC-MS/MS analysis of nascent peptide labeling by OPP in THUMPD1 WT and KO HEK-293T cells. Dashed line indicates log2FC and p-value < 0.05. Points corresponding to proteins significantly altered in the WT or KO samples are sized according to the number of peptide spectrum matches observed. Values are derived from n=3 biological replicates (Supplementary Data S12). (l) Comparison of summed ion intensity for proteins whose OPP-labeling was significantly altered between the HEK-293T THUMPD1 WT and KO samples. Significance was analyzed by two-tailed Student's *t* test (* = *P* < 0.05). (m) Single-cell RNA-Seq signature of THUMPD1 KO cells observed by Perturb-seq analysis of Replogle *et al* (46). (n) THUMPD1 KO cells do not show altered ability to activate mTOR signaling. Treatment conditions as follows: serum starved = removal of all amino acids and serum from medium (1 h), mTor inhibitor 1 = 1 μM AZD2014 (1 h), mTor inhibitor 2 = 10 nM rapamycin (1 h). Data are representative of n=2 biological replicates.

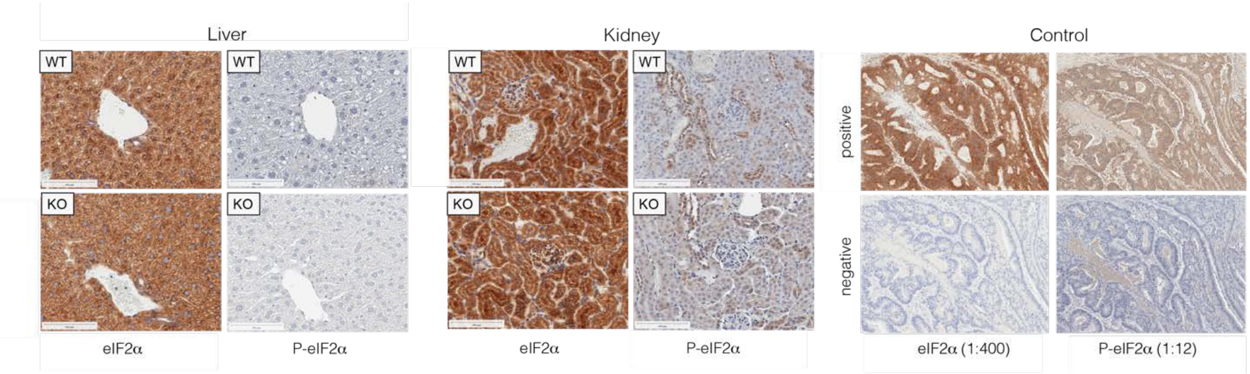

Figure S8. THUMPD1 knockout elevates eIF2α phosphorylation in mouse tissues. Immunohistochemical (IHC) staining of total eIF2α (left) and (Ser^{50}) P-eIF2α (right) in liver tissue and kidney tissue isolated from age-matched WT and *Thumpd1*$^{-/-}$ KO mice. Results are representative of n=4 biological replicates.

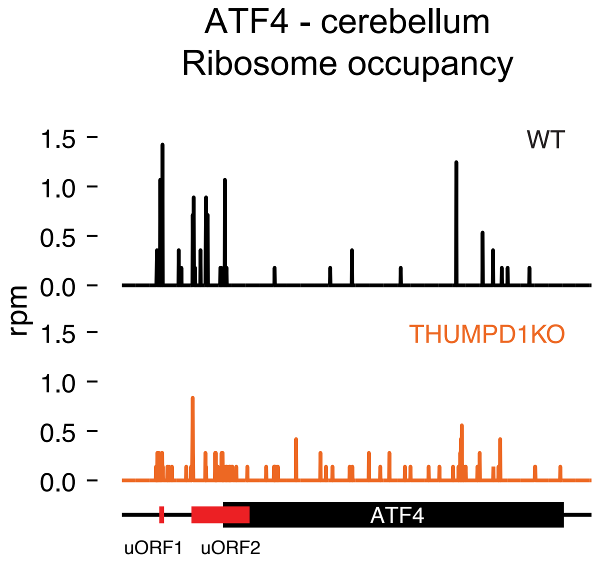

Figure S9. ATF4 ribosome occupancy is altered in THUMPD1-deficient cerebellum. Ribosome footprints in Atf4 gene from WT and THUMPD1 KO mouse cerebella. Red bars indicate the annotated uORFs of Atf4 gene. Elevated eIF2α phosphorylation in the THUMPD1KO background promotes translation of Atf4 main ORF.

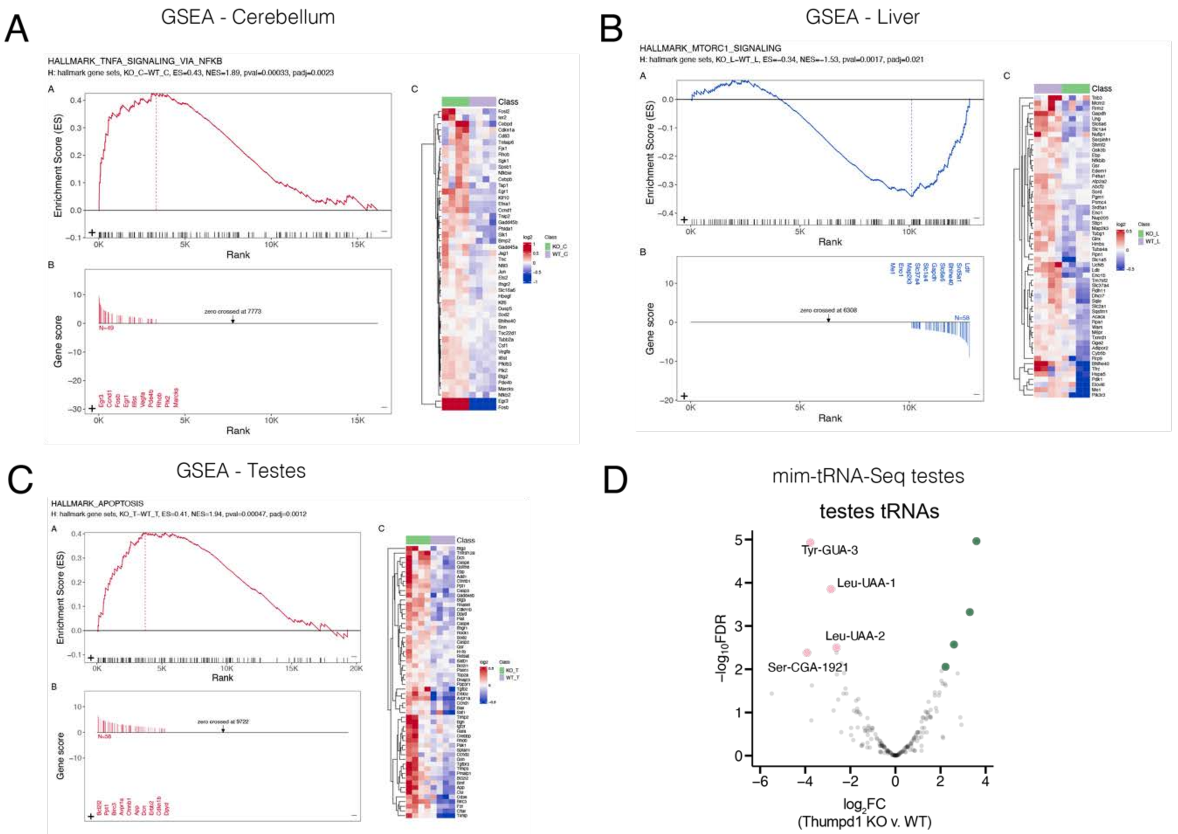

Figure S10. THUMPD1 loss triggers tissue-specific gene expression pathways. Gene Set Enrichment Analysis indicating activation of inflammatory gene expression in (a) mouse cerebellum (TNFA_SIGNALING_VIA_NFKB), (b) downregulation of transcripts associated with mTOR signaling (MTORC1_SIGNALING) in mouse liver, and (c) upregulated expression of apoptosis-associated genes in Thumpd1 KO testes. Pathway analyses were generated from RNA-Seq data (n=4 biological replicates). Additional pathway analyses are provided in Supplementary Data 9-11. (d) mim-tRNA-Seq analysis of WT v. THUMPD1 KO mouse testes indicates decreased levels of $tRNA^{Leu/Ser}$ isodecoders (Supplementary Data S7). Green points represent non-acetylated tRNA species that showed significant increases in abundance not marked by ac^4C. Data are derived from n=2 biological replicates for each tissue.

A

	Gcn2 WT			Gcn2 +/-			Gcn2 KO		
	Gcn2 WT, *Thumpd1 WT*	*Gcn2* WT, *Thumpd1$^{+/-}$*	*Gcn2* WT, *Thumpd1$^{-/-}$*	*Gcn2$^{+/-}$*, *Thumpd1 WT*	*Gcn2$^{+/-}$*, *Thumpd1$^{+/-}$*	*Gcn2$^{+/-}$*, *Thumpd1$^{-/-}$*	*Gcn2$^{-/-}$*, *Thumpd1 WT*	*Gcn2$^{-/-}$*, *Thumpd1$^{+/-}$*	*Gcn2$^{-/-}$*, *Thumpd1$^{-/-}$*
total	26	46	11	36	73	21	12	25	12
% actual	10%	18%	4%	14%	28%	8%	5%	10%	5%
% expected	6.25%	12.50%	6.25%	12.50%	25.00%	12.50%	6.25%	12.50%	6.25%

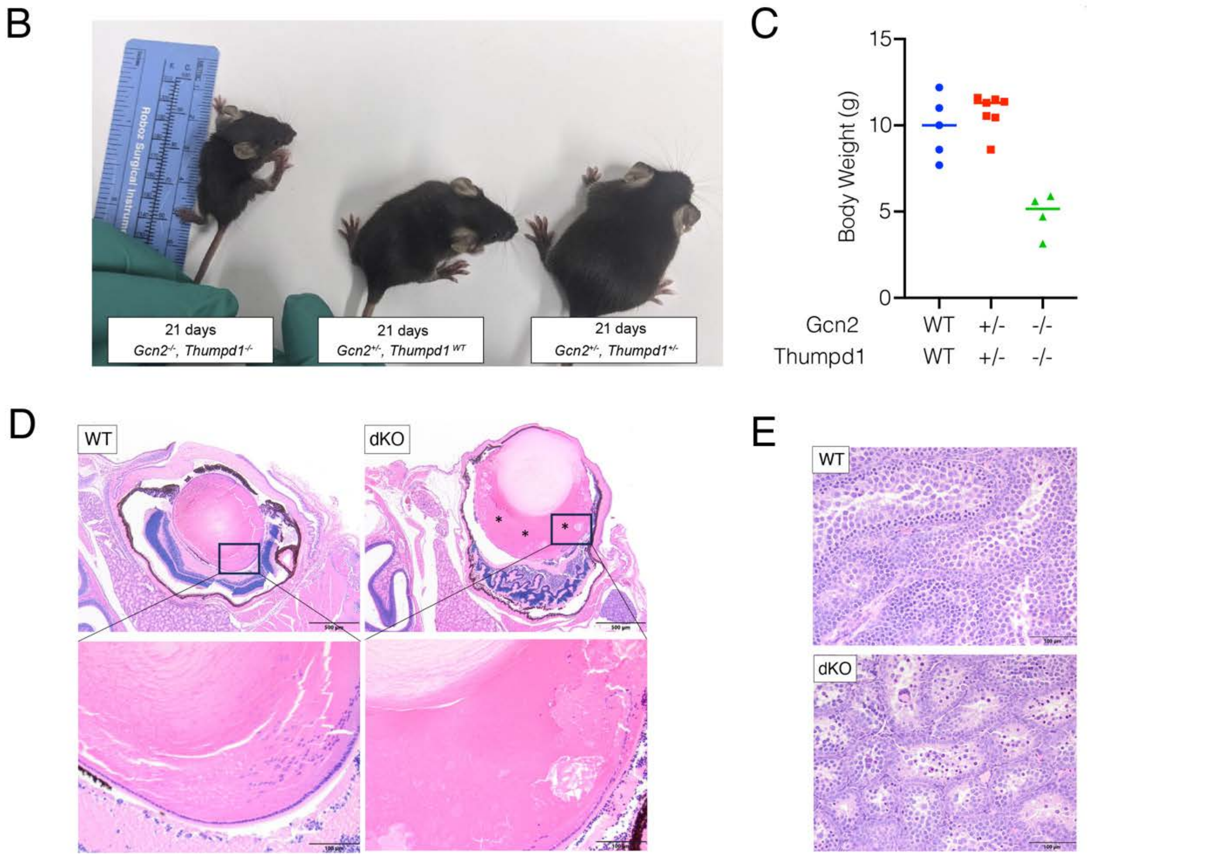

Figure S11. Combined loss of THUMPD1 and GCN2 influences offspring genotype and postnatal survival. (a) Offspring annotated by genotype produced by *Thumpd1$^{+/-}$*, *Gcn2$^{+/-}$* dihybrid cross. (b-c) *Thumpd1*/*Gcn2* double KO mice are runted. (d) A *Thumpd1$^{-/-}$*/*Gcn2$^{-/-}$* double knockout (DKO) animal exhibits cataractous change where the lens is expanded by a liquefaction of lens fibers (*), which lack organization and are swollen and fragmented, often forming globules of degenerate lens proteins (Morgagnian globules). Hyperplasia of the lens epithelium is also observed. The animal also exhibits retinal dysplasia, where the retina is disorganized, poorly developed, and thrown into folds. There is retinal detachment with hypertrophy (tomb-stoning) of the retinal pigmented epithelium. (e) *Thumpd1$^{-/-}$*, *Gcn2$^{-/-}$* DKO mice exhibit multifocal seminiferous tubule degeneration with syncytial cell formation. Due to the challenge of isolating *Thumpd1$^{-/-}$*, *Gcn2$^{-/-}$* DKO mice prior to lethality and autolysis onset, data for d-e are representative of n=1 individual mouse.

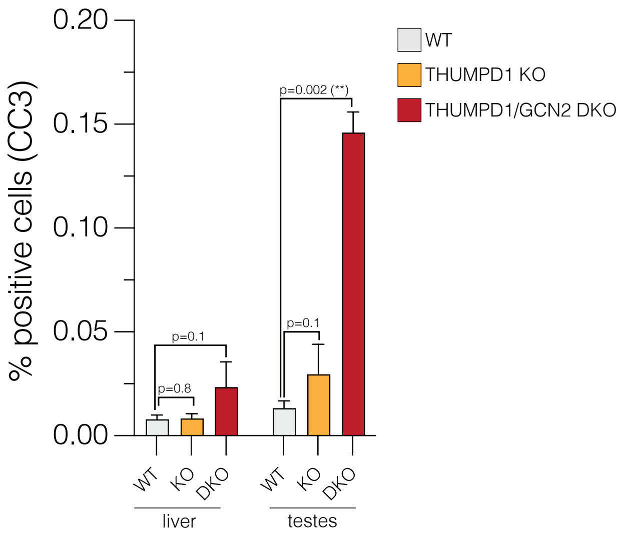

Figure S12. THUMPD1/GCN2 double knockout increases tissue apoptosis. Cleaved caspase 3 (CC3) staining of liver and testes tissue isolated from WT, Thumpd1 KO, and dual Thumpd1/Gcn2 DKO animals. Significance was analyzed by two-tailed Student's *t* test (ns = not significant, * = $P < 0.05$, ** = $P < 0.01$, and *** = $P < 0.001$). Data represent the average of n=3 (WT/KO) or n=2 (DKO) biological replicates.

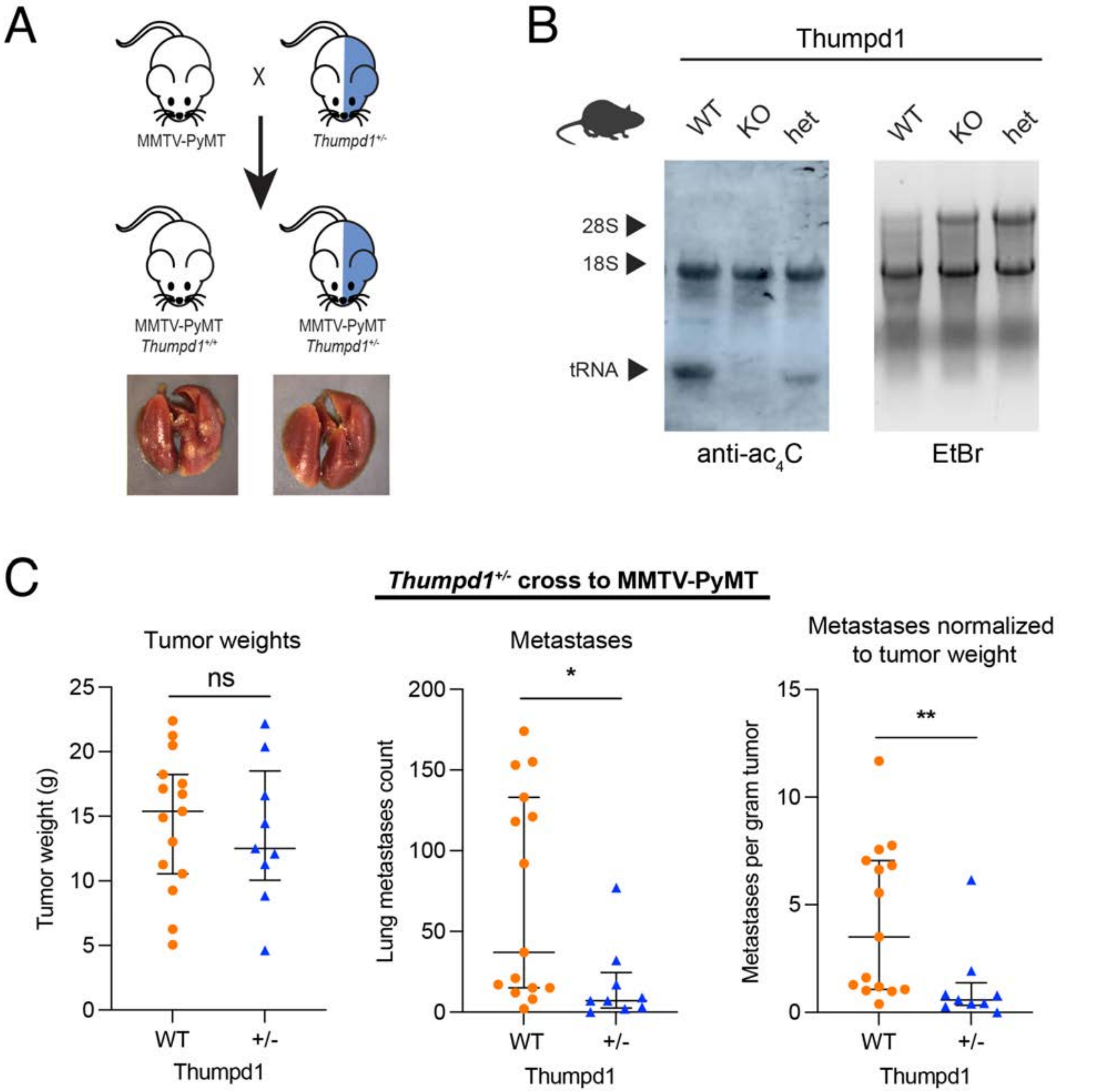

Figure S13. THUMPD1 haploinsufficiency suppresses breast cancer metastasis. (a) Schematic for PyMT tumor metastasis model. (b) *Thumpd1*$^{+/-}$ heterozygotes exhibit qualitatively decreased tRNA acetylation as assessed by immuno-Northern blot. (c) *Thumpd1*$^{+/-}$ heterozygosity suppresses breast cancer metastasis but not primary tumor growth in PyMT model. Significance was analyzed by two-tailed Student's t test (ns = not significant, * = $P < 0.05$, ** = $P < 0.01$, and *** = $P < 0.001$).

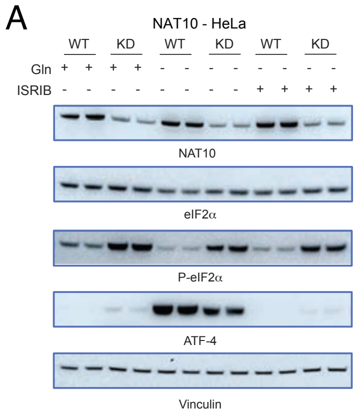

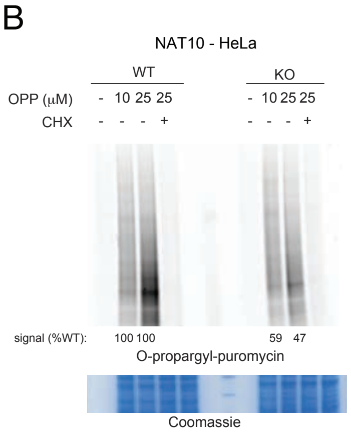

C

Cell line	OPP signal (% of WT)
HEK-293T THUMPD1 KO	80
HeLa THUMPD1 KO	67
HeLa Nat10 KD:	47

Figure S14. NAT10 depletion phenocopies THUMPD1 loss in translation regulation. (a) Analysis of eIF2α phosphorylation in NAT10 KD HeLa cells. Amino acid deprivation (-Gln) and ISRIB (1 μM) were used to induce translational stress. Biological replicates are loaded in adjacent lanes. (b) Analysis of global translation in NAT10 KD HeLa cells. O-propargyl puromycin (OPP) was used to label nascent transcripts, which were then ligated to a fluorophore-azide and visualized via SDS-PAGE. (c) Quantification of OPP signal in THUMPD1 KO and NAT10 KD cells as a percentage of WT OPP signal in the 25 μM OPP treated samples. Densitometry analysis was calculated using ImageJ software.

FULL UNCROPPED GELS AND WESTERN BLOTS

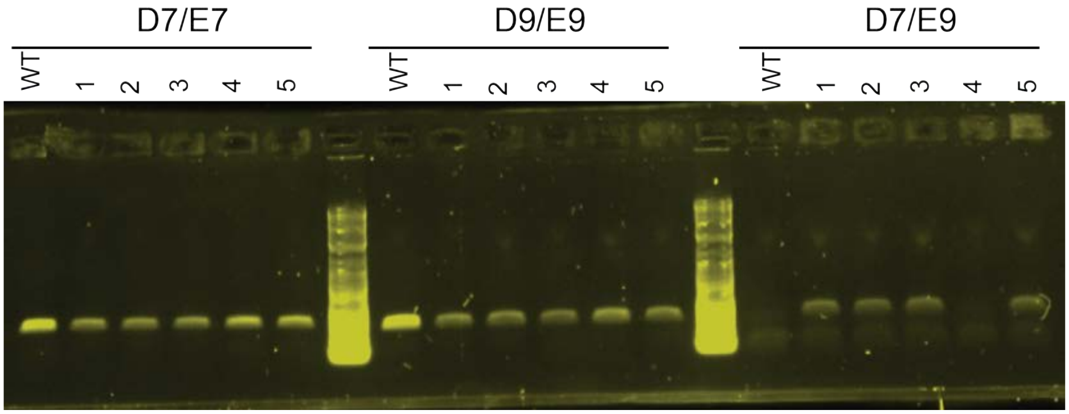

Full agarose gel image for PCR-based genotyping gel pertaining to Fig 1c. Lanes labeled as WT, 1, and 2 from each primer pair is shown in Fig 1c.

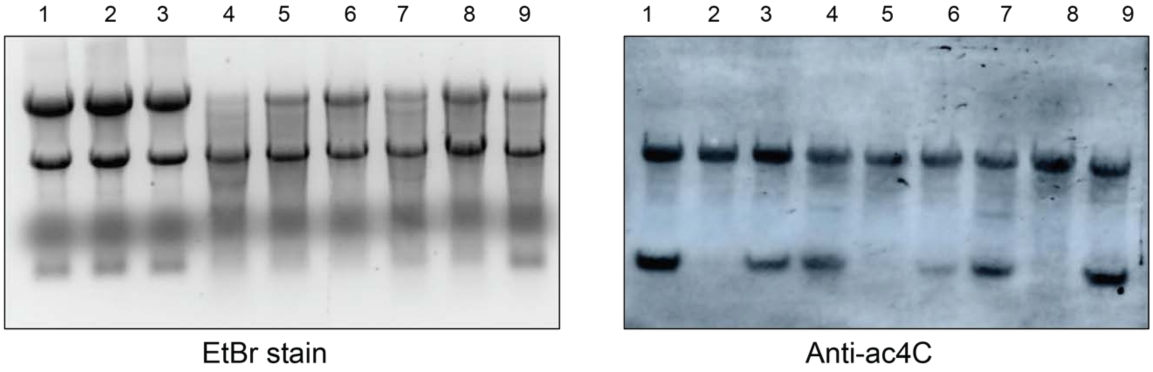

Full immuno-Northen blot and the Ethidium bromide-stained image pertaining to Fig 1d and Fig 3b. Lanes 4 and 5 correspond to data from mouse Thumpd1 WT and KO RNA shown in Fig 1d. Lanes 1 and 2 correspond to data from HEK-293T THUMPD1 WT and KO RNA shown in Fig 3b.

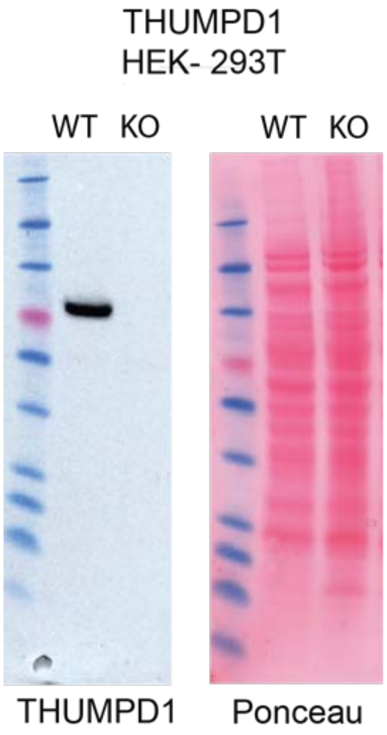

Full western blots pertaining to Fig 3a.

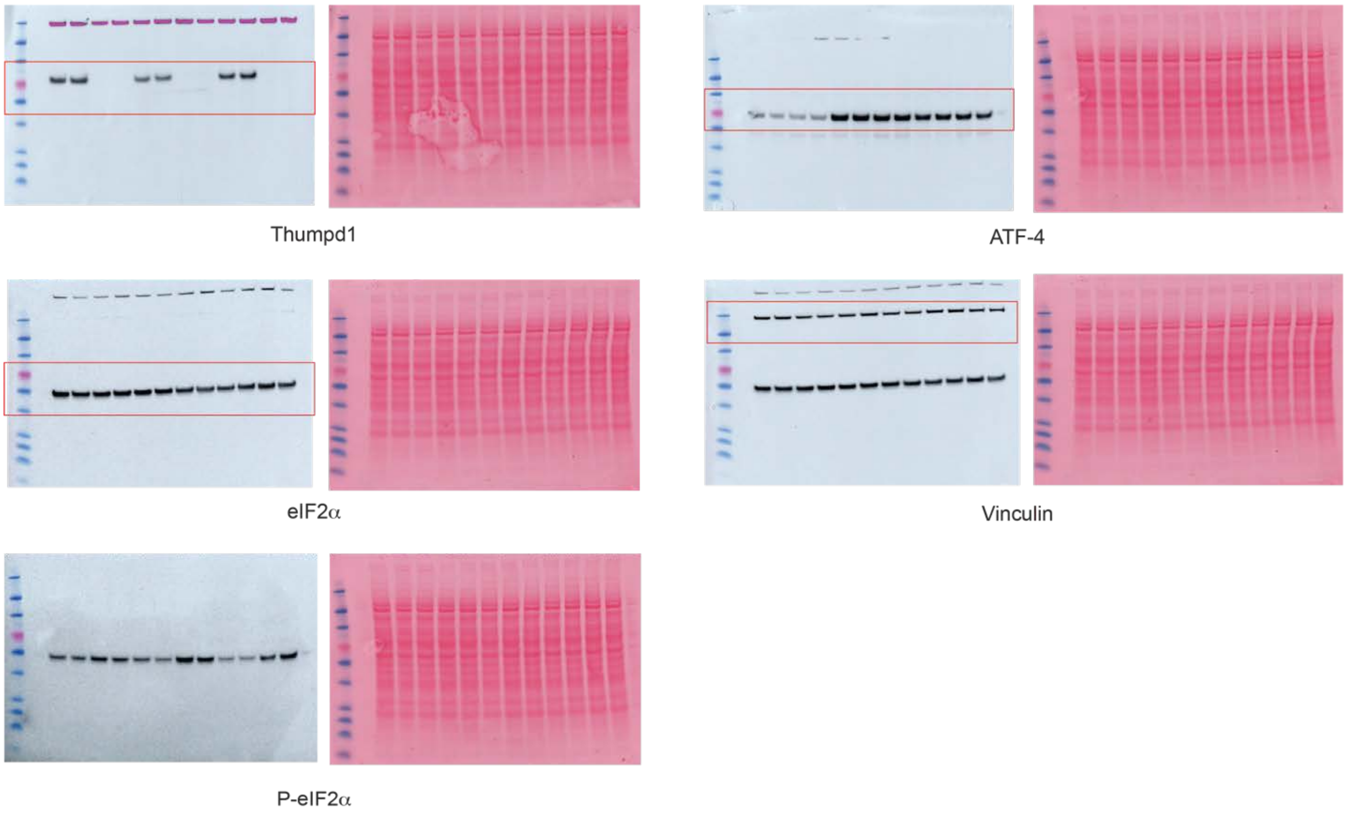

Full Western blots pertaining to Fig 4e.

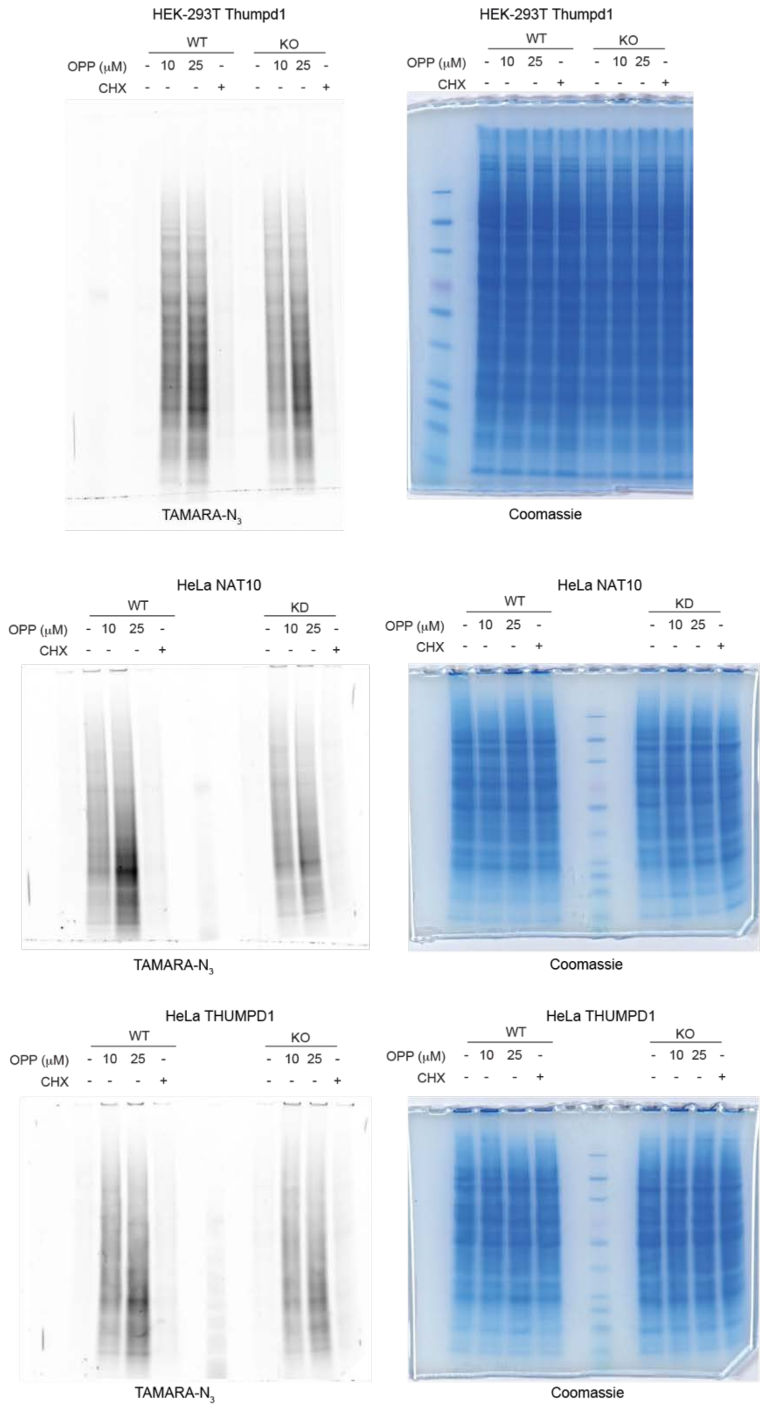

Full SDS-PAGE gels pertaining to Fig 4f, S7j, and S14b.

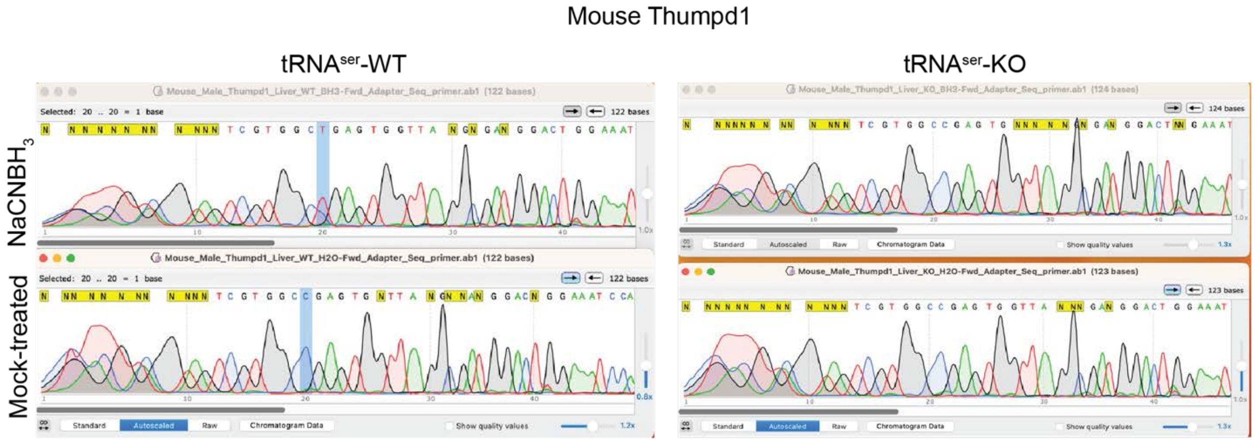

Full Sanger traces pertaining to Fig S1f.

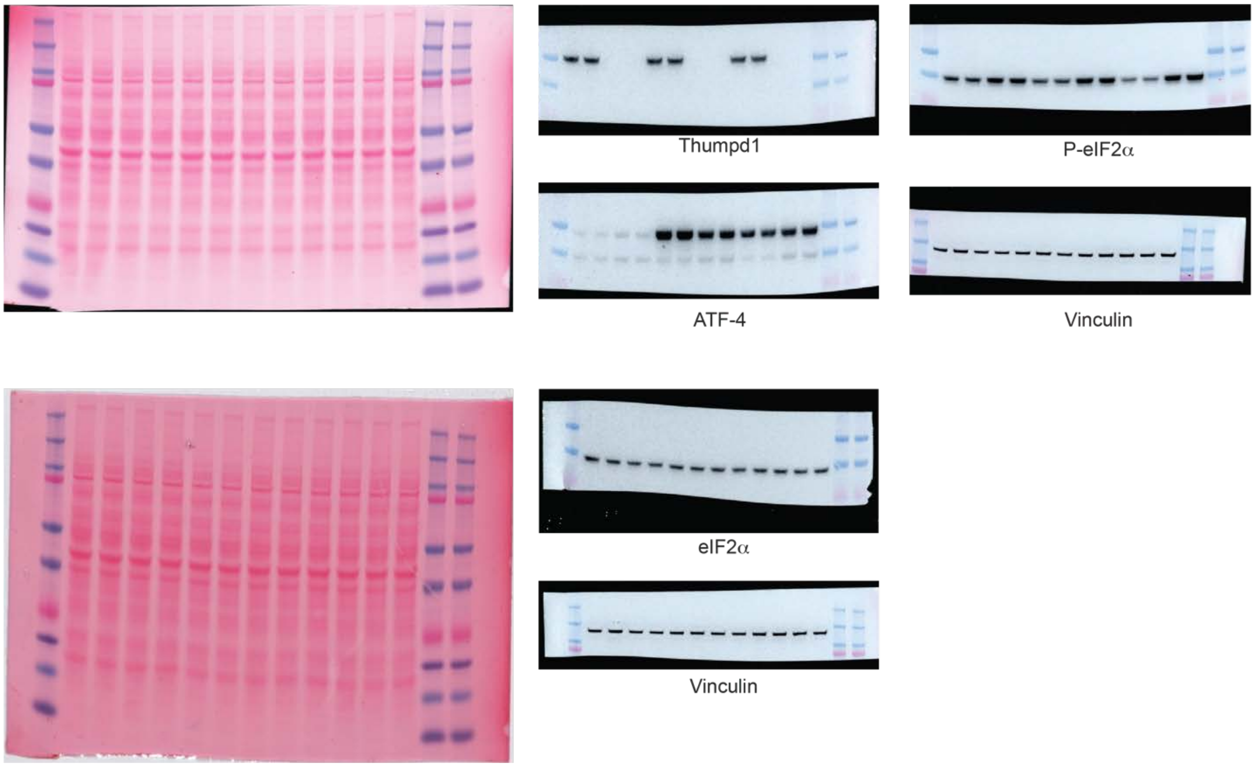

Full Western blots pertaining to Fig S7c. Corresponding Ponceau staining is shown on the left side of the blots.

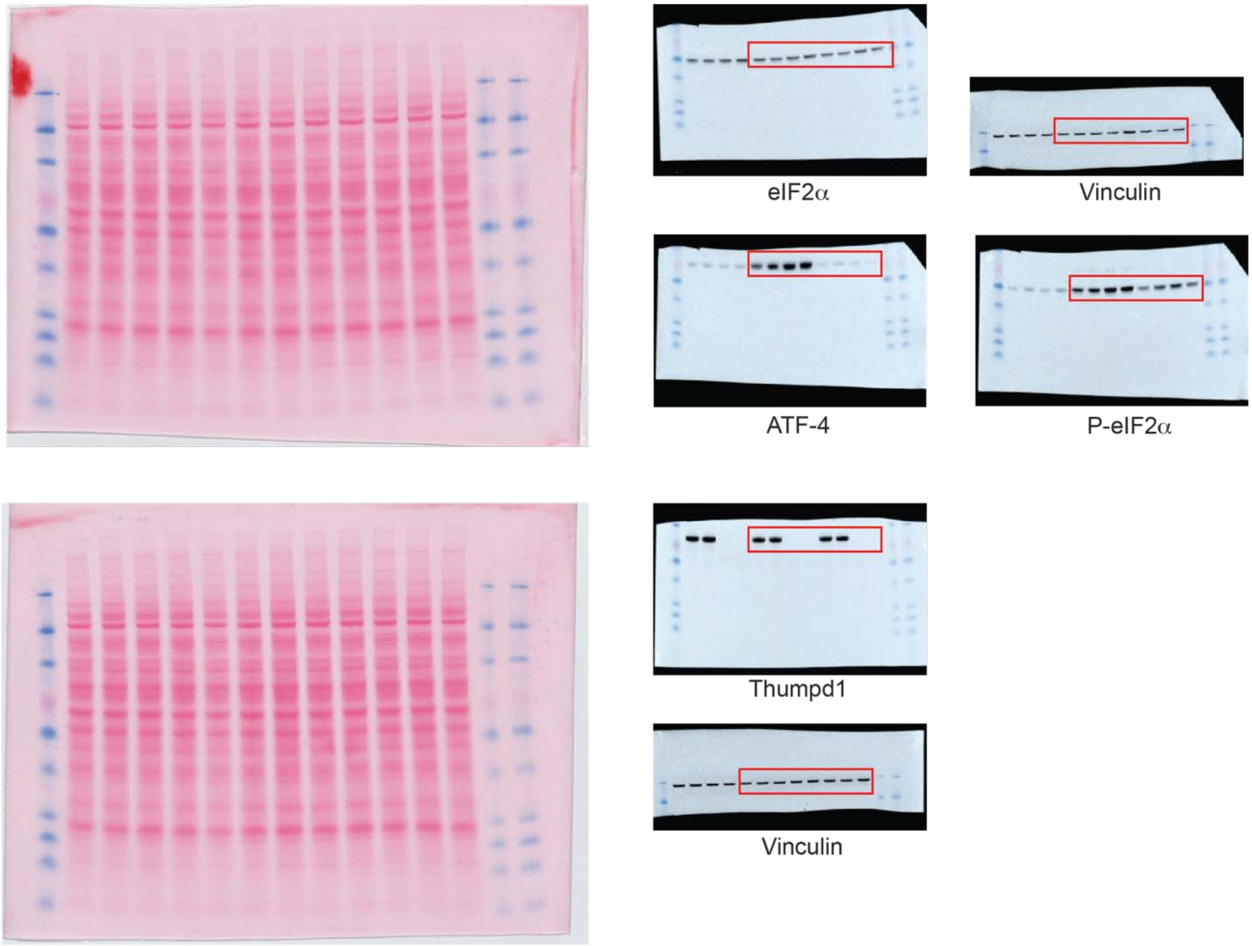

Full Western blots pertaining to Fig S7d. Corresponding Ponceau staining is shown on the left side of the blots.

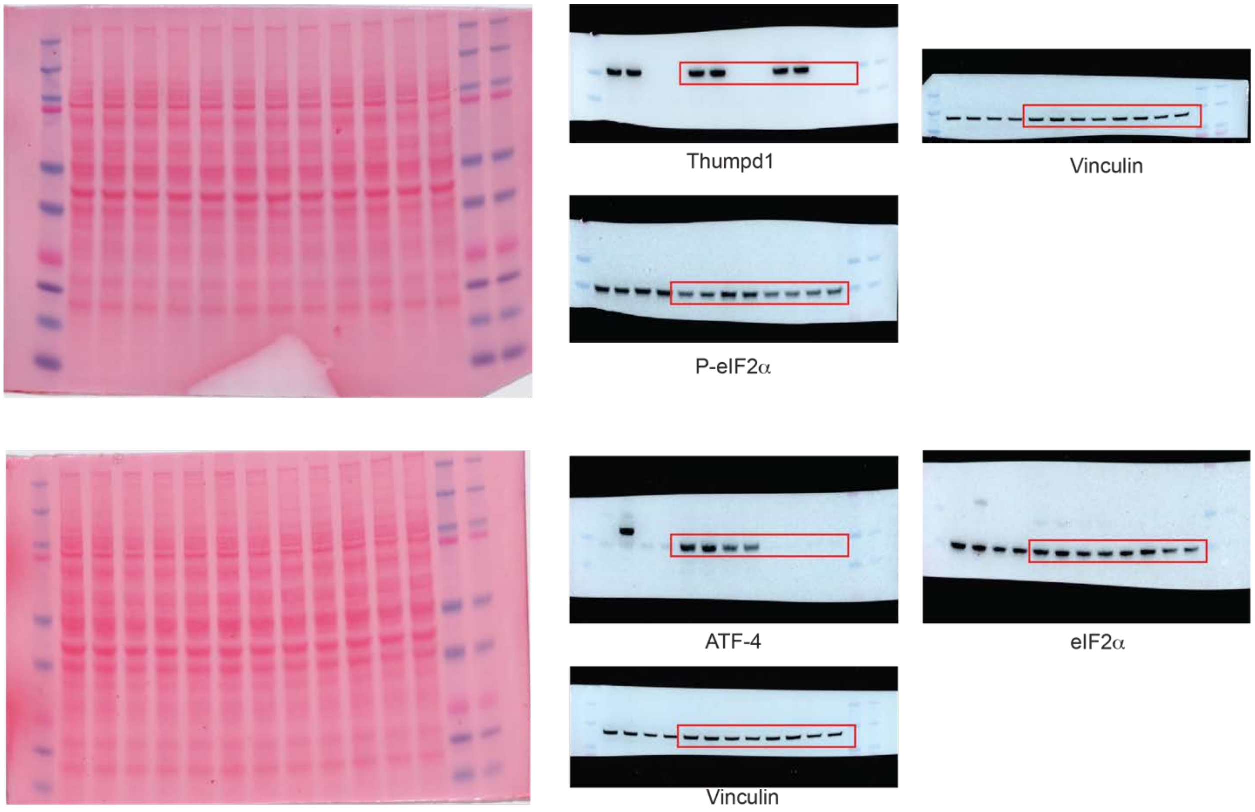

Full Western blots pertaining to Fig S7e. Corresponding Ponceau staining is shown on the left side of the blots.

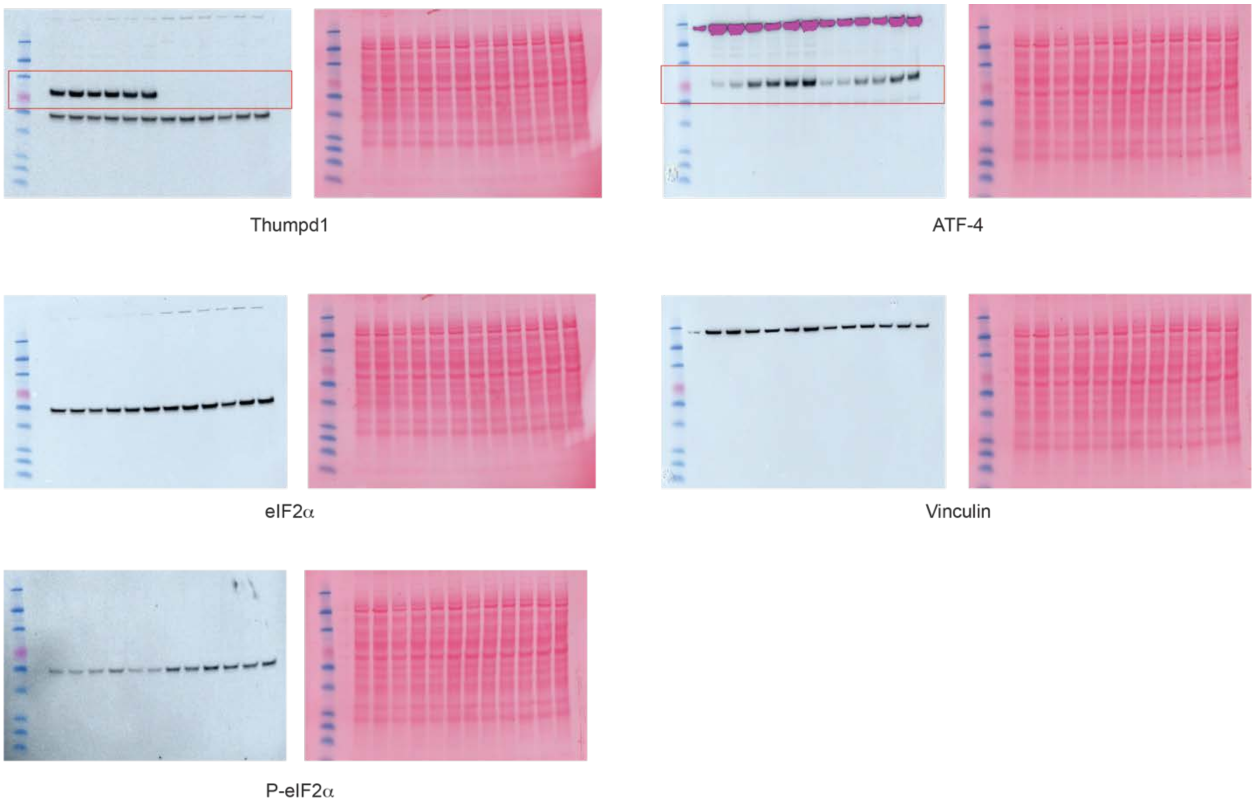

Full Western blots pertaining to Fig S7h.

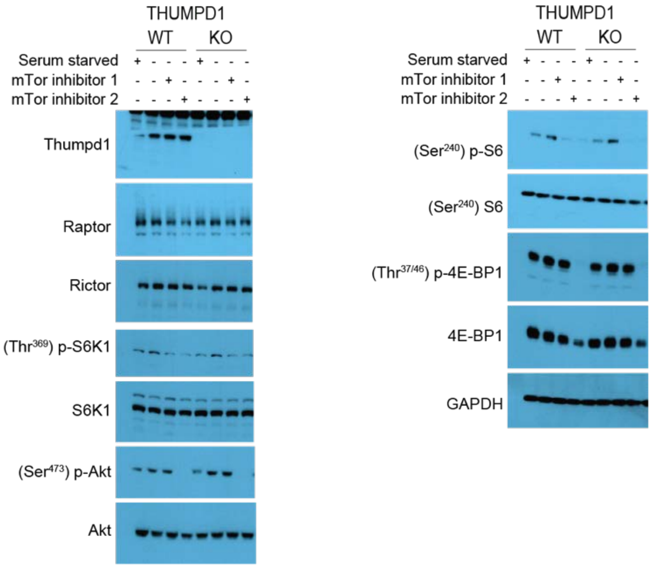

Autoradiographs pertaining to Fig S7n.

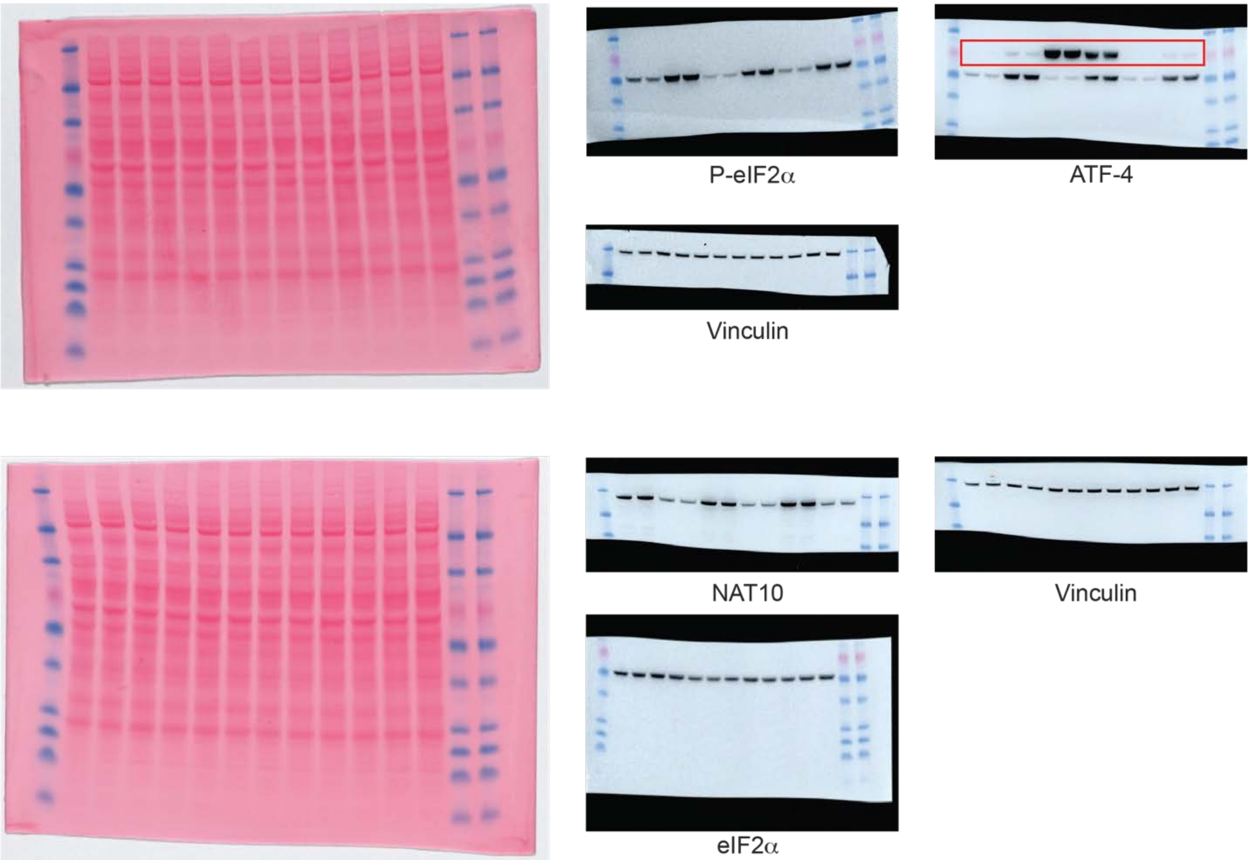

Full Western blots pertaining to Fig S14a. Corresponding Ponceau staining is shown on the left side of the blots.

LEGENDS FOR ADDITIONAL SUPPLEMENTARY FILES

Table S1. Antibodies, Software and algorithms used in this study

Table S2. Oligonucleotide sequences used in this study

Data S1. ac^4C-seq analysis of tRNA acetylation sites across eukaryotic models

Data S2. mim-tRNA-Seq quantification of tRNA levels in THUMPD1 WT and KO HEK-293T cells

Data S3. Ribosome profiling analysis of codon occupancy in THUMPD1 WT and KO HEK-293T cells

Data S4. Proteomic analysis of THUMPD1 WT and KO HEK-293T cells

Data S5. Codon usage analysis of transcripts with altered translational efficiency in THUMPD1 KO cells

Data S6. Disome profiling analysis of ribosome collisions in THUMPD1 WT and KO HEK-293T cells

Data S7. mim-tRNA-Seq quantification of tRNA levels in cerebellum, liver and testes of WT and THUMPD1 KO mice

Data S8. Ribosome profiling analysis of ribosome collisions in THUMPD1 WT and KO mice cerebella

Data S9. Differential gene expression analysis of cerebella from WT and THUMPD1 KO mice

Data S10. Differential gene expression analysis of liver from WT and THUMPD1 KO mice

Data S11. Differential gene expression analysis of testes from WT and THUMPD1 KO mice

Data S12. LC-MS/MS analysis of nascent peptide labeling by OPP in THUMPD1 WT and KO HEK-293T cells
